# Modeling Drug Responses and Evolutionary Dynamics Using Patient-Derived Xenografts Reveals Precision Medicine Strategies for Triple-Negative Breast Cancer

**DOI:** 10.1158/0008-5472.CAN-24-1703

**Published:** 2024-11-08

**Authors:** Abigail Shea, Yaniv Eyal-Lubling, Daniel Guerrero-Romero, Raquel Manzano Garcia, Wendy Greenwood, Martin O’Reilly, Dimitra Georgopoulou, Maurizio Callari, Giulia Lerda, Sophia Wix, Agnese Giovannetti, Riccardo Masina, Elham Esmaeilishirazifard, Wei Cope, Alistair G. Martin, Ai Nagano, Lisa Young, Steven Kupczak, Yi Cheng, Helen Bardwell, Elena Provenzano, Justine Kane, Jonny Lay, Louise Grybowicz, Karen McAdam, Carlos Caldas, Jean Abraham, Oscar M. Rueda, Alejandra Bruna

**Affiliations:** 1 Department of Oncology, Cancer Research UK Cambridge Institute, Li Ka Shing Centre, University of Cambridge, Cambridge, United Kingdom.; 2 Institute of Cancer Research, London, United Kingdom.; 3 Fondazione Michelangelo, Milan, Italy.; 4 Breast Cancer Now Toby Robins Research Centre, The Institute of Cancer Research, London, United Kingdom.; 5 Clinical Genomics Unit, Fondazione IRCCS Casa Sollievo della Sofferenza, Foggia, Italy.; 6 Department of Histopathology, Cambridge University NHS Foundation Trust, Cambridge, United Kingdom.; 7 Cambridge NIH Biomedical Research Centre, Cambridge, United Kingdom.; 8 Department of Oncology, Precision Breast Cancer Institute, University of Cambridge, Cambridge, United Kingdom.; 9 Cambridge University NHS Foundation Trust, Cambridge, United Kingdom.; 10 Department of Clinical Biochemistry, Institute of Metabolic Science, University of Cambridge, Cambridge, United Kingdom.; 11 MRC-Biostatistics Unit, University of Cambridge, Cambridge, United Kingdom.

## Abstract

The intertumor and intratumor heterogeneity of triple-negative breast cancers, which is reflected in diverse drug responses, interplays with tumor evolution. In this study, we developed a preclinical experimental and analytical framework using patient-derived tumor xenografts (PDTX) from patients with treatment-naïve triple-negative breast cancers to test their predictive value in personalized cancer treatment approaches. Patients and their matched PDTXs exhibited concordant drug responses to neoadjuvant therapy using two trial designs and dosing schedules. This platform enabled analysis of nongenetic mechanisms involved in relapse dynamics. Treatment resulted in permanent phenotypic changes, with functional and therapeutic consequences. High-throughput drug screening methods in *ex vivo* PDTX cells revealed patient-specific drug response changes dependent on first-line therapy. This was validated *in vivo*, as exemplified by a change in olaparib sensitivity in tumors previously treated with clinically relevant cycles of standard-of-care chemotherapy. In summary, PDTXs provide a robust tool to test patient drug responses and therapeutic regimens and to model evolutionary trajectories. However, high intermodel variability and permanent nongenomic transcriptional changes constrain their use for personalized cancer therapy. This work highlights important considerations associated with preclinical drug response modeling and potential uses of the platform to identify efficacious and preferential sequential therapeutic regimens.

**Significance:**

Patient-derived tumor xenografts from treatment-naïve breast cancer samples can predict patient drug responses and model treatment-induced phenotypic and functional evolution, making them valuable preclinical tools.

## Introduction

Breast cancers exhibit extensive intertumor and intratumor heterogeneity at both the genomic and phenotypic levels. This is reflected in the diversity of interpatient drug responses, clinical prognoses, and patterns of relapse (1–9). Patient-derived tumor xenografts (PDTX) have been shown to retain both interpatient diversity and intratumor heterogeneity and offer an unprecedented opportunity to study human breast cancer at a high resolution (10–18). We have previously generated and molecularly characterized a living biobank of breast cancer PDTXs and demonstrated that these recapitulate the main histologic and genomic features of the originating tumor, including intratumor heterogeneity (19). In addition, we pioneered the use of dissociated PDTX cells) for high-throughput drug screening, with most drug responses validated *in vivo*. We developed a mass cytometry antibody panel tailored to breast cancer PDTXs, allowing highly resolved multiparametric single-cell phenotyping and its impact on drug responses (20).

PDTX and PDTX cell drug response profiling offer exciting opportunities as anticipatory tools for personalized medicine. However, the extent to which PDTXs mirror therapy responses of the matched donor patient has been insufficiently studied. This is further complicated when patients receive multiple lines of therapy prior and subsequent to the development of the model. Indeed, although there is strong evidence that drug pressure (such as *in vivo* treatment) impacts the phenotypic evolution of a PDTX tumor (20, 21), its functional implications are rarely acknowledged in preclinical studies. Subsequent lines of clinical therapy could impede accurate drug response profiling when PDTXs are generated early in the disease trajectory. However, little evidence has been generated to directly test this, and in this study, we aimed to resolve this disparity. In addition, the heterogeneity observed between PDTX models, such as growth dynamics, urges a robust assessment of different *in vivo* trial designs and analytical approaches to determine the impact on patient–PDTX drug responses.

In this study, we developed a preclinical platform to robustly and systematically assess the concordance of patient–PDTX drug responses using eight treatment-naïve PDTX models and test efficacious treatment strategies for each patient. This was aligned with a prospective neoadjuvant clinical trial for patients with triple-negative breast cancer (TNBC) and/or *BRCA1/2* alterations. We then examined the impact of *in vivo* treatment on a tumor’s phenotypic evolution and its functional consequences. These data demonstrate the potential utilities of PDTXs as anticipatory tools and highlight crucial considerations to be addressed when using PDTX models for preclinical drug response profiling.

## Materials and Methods

### Generation and maintenance of PDTXs

Breast cancer PDTXs were established and passaged as described in ref. 19. Patient primary breast cancer biopsy samples were collected at diagnosis and immediately transported to the animal facility. Patient tissue samples were embedded in Matrigel and implanted subcutaneously into ∼2 female severely immunocompromised NOD.Cg-Prkdcscid Il2rgtm1Wjl/SzJ (NSG) mice (RRID: BCBC_4142). PDTXs were serially implanted into multiple hosts to allow for *in vivo* expansion of each model. Xenograft samples were flash-frozen in liquid nitrogen, cryopreserved in freezing media (FBS/10% DMSO), and fixed in 10% neutral buffered formalin at each passage from each mouse. Regular genotyping and IHC quality control was performed routinely to confirm the propagation of human breast cancer tissue matching the originating patient-derived sample.

All use of human samples and PDTX generation is covered by the appropriate human ethics framework in the United Kingdom. The research was done with the appropriate ethical approval, and written informed consent was given by all patients included in this study. In addition, consent is defined by the NIH and National Institute for Health and Care Research as not simply a signed piece of paper; it is defined as an ongoing process that includes knowledge of use of patient data and clinical samples (where relevant). PARTNER samples and PDTX models derived from PARTNER samples were collected and held under the PARTNER trial ethics: IRAS project ID 178681. The PARTNER trial protocol (NCT03150576) was approved by the North West – Haydock Research Ethics Committee (reference number: 15/NW/0926), and the trial was carried out in accordance with the Declaration of Helsinki and the European Clinical Trials Directive 2001/20/EC. ClinicalTrials.gov Identifier: NCT03150576. The Personalized Breast Cancer Program was approved by the Cambridge Central Research Ethics Committee (reference number: 18/EE/0251).

All animal work was approved and conducted under the UK Home Office regulatory framework (project license number: P1266F82E) and was approved by the Institutional Ethics Committee.

### *In vivo* preclinical drug studies

Fresh or cryopreserved tumor fragments from a single mouse were passaged into a cohort of NSG mice following statistical power analysis. At the appropriate point [either a defined time after implantation or based on tumor volume (TV)], mice were enrolled into treatment cohorts and dosed according to the allocated treatment schedules. For trial design 2, in which treatment started at a fixed TV, mice were assigned into cohorts using a stratified randomization approach, which aims to evenly distribute initial TV sizes. To achieve this, TVs from a group of mice are ranked from low to high and then assigned to a cohort using a spiral approach (e.g., 1–3, 3–1, 1–3… for three cohorts).

For chemotherapy (paclitaxel and carboplatin; CT) and CT (paclitaxel and carboplatin) plus olaparib (CTO) trial arms, four cycles of therapy were administered (3 weeks per cycle) to total 11 weeks of treatment. Carboplatin was administered by i.v. injection once every 3 weeks (day 1 of each cycle with 0.16 mg and 40 mg/kg doses for trial designs 1 and 2, respectively). Paclitaxel was administered by i.v. injection once weekly (days 1, 8, and 15 of each cycle with doses of 0.07 mg and 7 mg/kg for trial designs 1 and 2, respectively). Olaparib was administered by oral gavage on days 3 to 14 of each cycle (0.05 mg or 50 mg/kg for trial designs 1 and 2, respectively). For olaparib monotherapy, olaparib (50 mg/kg) was administered by oral gavage with 5 days on/2 days off for 11 weeks. For AZD1775 monotherapy, AZD1775 (120 mg/kg) was administered by oral gavage with 5 days on/2 days off for 11 weeks. For olaparib /AZD1775 combination, models 1006 and 1040 were administered with olaparib (50 mg/kg) 5 days on/2 days off and AZD1775 120 mg/kg 5 days on/2 days off. Models 1008, 1022, 1053, and 1141 were administered with olaparib (50 mg/kg) daily and AZD1775 (60 mg/kg) 3 days on/4 days off.

Paclitaxel (Selleckchem, S1150) was formulated in 1:1 ethanol:Kolliphor (Sigma, C5135-500G) and diluted prior to use in Vetivex saline. Carboplatin (Selleckchem, S1215) was formulated in sterile water. Olaparib (provided by AstraZeneca) was formulated in 30% Kleptose/10% DMSO in water, and AZD1775 (provided by AstraZeneca) was formulated in 0.5% methylcellulose. Mice were carefully monitored for adverse effects, and tumors were measured weekly with calipers. Mice were humanely sacrificed at the defined endpoint (either immediately after treatment or at TV 1,500 mm^3^). All *in vivo* drug trials were performed at Cancer Research UK Cambridge Institute’s animal facility.

For the preclinical trial in which sequential CT and olaparib treatment was tested, cryopreserved tumor fragments from model 1040 (untreated or previously treated using trial design 1 with CT or olaparib) were reimplanted into 15 to 18 NSG mice. For each group, when tumors reached ∼200 mm^3^, mice were assigned into untreated, CT, or olaparib cohorts using a stratified randomization approach, as described above. CT cohorts were administered with carboplatin (0.16 mg) once every 3 weeks (day 1 of each cycle) and paclitaxel (0.07 mg) once per week (days 1, 8, and 15 of each cycle). Both carboplatin and paclitaxel were administered by i.v. injection for a total of 11 weeks of treatment (four cycles). Olaparib cohorts were treated with olaparib (50 mg/kg) by oral gavage with 5 days on/2 days off for 11 weeks.

### Measurement of TV

Calipers were used to measure the volume of the mouse tumors by using the height (*h*) and width (*w*) weekly. The TV (mm^3^) was determined by using the following equation:

$$TV=\frac{\left(W^{2}\right)H}{2}$$

### PDTX tumor growth and drug response modeling (coclinical trials)

For each preclinical trial, we conducted a series of analyses to assess the drug response effects in various PDTX models. We fitted a single linear mixed-effects model for each unique PDTX model to estimate the drug response effect. The log-transformed TV was modeled as a function of time, treatment group, and their interaction, with random intercepts and slopes for each mouse using the R packages lme4 (RRID: SCR_015654) and lmerTest (RRID: SCR_015656).

Four metrics were derived from the fitted models to quantify treatment effects:

i. Interaction between treatment and time, relative to the untreated group (change in log-growth rate): Estimates the difference in tumor growth rates between each treatment group and the untreated group.
ii. Estimated difference in the AUC between the treatment cohort and the untreated cohort: Compares the overall tumor burden over the entire study period between each treatment cohort and the untreated cohort. This is computed using the estimated fixed effects (i.e., with the overall mean of the treatment group and not on each individual mouse).
iii. Sum of main and interaction effects for each treatment group (log-growth rate under treatment): Estimates the log-daily growth rate under each treatment, combining the main effect of time and the interaction between time and treatment.
iv. Predicted TVs at the end of treatment: Compares the predicted TVs at the end of the study period between treatment groups.

Note that the first three metrics produce a single value, whereas the fourth produces a value for each mouse, as it takes into account individual mouse variability through the random effects.

PDTX models were assigned into two response groups based on the matched patient’s clinical response, as determined at surgery following neoadjuvant treatment. Pathologic complete response (pCR) and non-pCR were used to classify patients. To assess the relationship between *in vivo* treatment response and pCR status, we performed Wilcoxon rank-sum tests for each metric, comparing the estimates between the pCR and non-pCR groups.

Trial design 1 was tested in six unique PDTX models, whereas trial design 2 was tested in eight unique PDTX models. The same analytical approach and statistical tests were applied to both trials during the phase of treatment, providing a comprehensive evaluation of drug response effects and their relationship with pCR status in the PDTX models. Given that no apparent violations of the model assumptions were observed, the selected models were carried forward. The *P* values were further subjected to multiplicity correction adjustments to account for the within-patient analyses and comparisons conducted. For the third metric related to the change in growth rate under treatment, we estimated the statistical significance using the variance-covariance matrix from the mixed-effects model, degrees of freedom, fixed effects, and the SE to construct the t-statistic and corresponding confidence intervals.

### PDTX drug response modeling (model 1040 treated sequentially with no treatment, CT, or olaparib)

We conducted a 3 × 3 factorial analysis to assess the impact of previous and current treatments on tumor growth in the PDTX model 1040. Tumors were initially treated with either no treatment (untreated), CT, or olaparib. After harvesting, tumors from each previous treatment group were reimplanted into mice and randomized to receive either no treatment (untreated), CT, or olaparib.

For each second-line treatment, a linear mixed-effects model was fitted using the first-line treatment as a covariate. The log-transformed TV was modeled as a function of time, first treatment, and their interaction, with random intercepts and slopes for each mouse.

We used the four mathematical modeling metrics as described above.

### DNA/RNA extraction from PDTX tumors

PDTX tissue samples were flash-frozen in liquid nitrogen. Tissue samples were homogenized using a tissue lyser (Qiagen). DNA and RNA for bulk RNA sequencing (RNA-seq) and whole-exome sequencing (WES) was extracted using AllPrep DNA/RNA Kit (Qiagen) following the manufacturer’s instructions.

### Bulk RNA-seq

RNA-seq libraries for Illumina sequencing were prepared using TruSeq Stranded mRNA Kit (Illumina, 20020595) according to the manufacturer’s instructions for high-throughput sample workflow. An input of 500 ng of total RNA per sample was used for library preparation. After DNA fragments were enriched by PCR, all libraries were quantified using KAPA Library Quantification Kit (KAPA Biosystems) and analyzed by TapeStation (Agilent). Successful libraries were normalized based on concentration and pooled. Libraries were sequenced on either HiSeq 4000 or NovaSeq 6000 sequencing platforms (Illumina) according to the manufacturer’s instructions. Sequencing was performed using either 50 bp single-end reads or 50 bp paired-end reads to generate on average 15 million total reads per library. The bioinformatics analysis of the RNA-seq data was performed using R 4.0.3. Gene Set Enrichment Analysis (GSEA) was done in single-sample mode (ssGSEA) with the GSVA package (RRID: SCR_021058) and fgsea package (RRID: SCR_020938) for pathway analysis. Normalization and differential gene expression analysis was done using edgeR package (RRID: SCR_012802).

### WES

WES libraries were prepared using Nextera Flex for Enrichment (Illumina, 20025524) according to the manufacturer’s instructions. Libraries were sequenced on NovaSeq 6000 using 100 bp paired-end reads, aiming for approximately 100× coverage. Reads were aligned using novoAlign (Novocraft, RRID: SCR_014818) applying our In silico Combined human-mouse Reference Genome (ICRG) pipeline to remove mouse contamination (22). BAM files were merged, sorted, and indexed using samtools (RRID: SCR_002105). Duplicates were marked using Picard tools (RRID: SCR_006525). Candidate single-nucleotide variants (SNV) and indels were called using GATK HaplotypeCaller (RRID: SCR_001876). Variants were annotated using Annovar (version 2018-04-16, RRID: SCR_012821) for gene/exon annotation, 1000 Genomes (version August 2015, RRID: SCR_006828), and dbSNP (single nucleotide polymorphism database; version SNP142, RRID: SCR_002338). Somatic mutations were identified by filtering out calls present in dbSNP and 1000 Genomes or in an internal panel of normal (*n* = 95). In the latter, variants were called with the same pipeline and reported if present in at least two samples.

### Preparation of single-cell suspensions from PDTX tumors

Cryopreserved PDTX tumor fragments were thawed rapidly into RPMI (Gibco, Invitrogen). Mechanical and enzymatic dissociation was performed using human Tumor Dissociation Kit (Miltenyi Biotec, 130-095-929) and gentleMACS Dissociator according to the manufacturer’s instructions for medium tumors. After tissue dissociation, single-cell suspensions were strained through 70-μm meshes (BD Biosciences) and transferred into RPMI (Gibco, Invitrogen).

### Single-cell RNA-seq

Single-cell suspensions of PDTX tumors were prepared as described above. Single-cell sequencing was performed using Chromium Single Cell 3′ GEM, Library and Gel Bead Kit v2 (10x Genomics, PN-120237) and Chromium Single Cell A Chip Kit (10x Genomics, PN- 120236) according to the manufacturer’s instructions. Libraries were sequenced by HiSeq2500 (Illumina) or NovaSeq 6000 SP or S1 flow cells (Illumina) with paired-end reads and dual-indexing. Cell Ranger v5.0 (10x Genomics) was used to generate raw count matrices with the hgmm (human genome mus musculus) reference. CellBender (23) was used to remove ambient noise and generate the count matrices for the filtered cells. Cells with at least 750 unique molecular identifiers (UMI) from nonmitochondrial genes and less than 40% UMIs from mitochondrial genes were kept for analysis. Next, Souporcell (24) was used with *K* = 2 to cluster cells into human and mouse clusters and to remove doublets. Cells in the human cluster with the majority of their UMIs coming from human genes were kept. Finally, Scrublet (25) was used with 0.3 as a cut-off for doublet score to further remove potential doublet cells.

### Single-cell RNA-seq metacell analysis

To select feature genes for metacell (MC) analysis, we first identified high-variance and strong genes and then removed mitochondrial genes, a few strong noncoding genes, and gene modules correlated with cell cycle, IFN, and stress responses (Supplementary Table S1). This resulted in a set of 3,390 feature genes. MCs were derived as described (26) using *K* = 110, standard bootstrapping, and MC splitting. We further filtered two MCs suspected to be nuclei stripped from their cytoplasm as they had high fraction of UMIs from long noncoding RNA and mitochondrial genes, low number of UMIs, and low fraction of UMIs from ribosomal genes. We also filtered out a single MC suspected to contain epithelial–mesenchymal (EM) doublet cells. The derived final model included 21,087 cells partitioned into 153 MCs.

### Western blot

Flash-frozen PDTX tissue samples were homogenized using a tissue lyser (Qiagen) in protein lysis buffer: Tris-HCl (50 mmol/L), sodium chloride (150 mmol/L), Triton X-100 (1%), EDTA (5 mmol/L), sodium fluoride (50 mmol/L), β-glycerophosphate (25 mmol/L), protease inhibitors (Thermo Fisher Scientific, 1861279), and phosphatase inhibitors (Thermo Fisher Scientific, 78427). Tissue was incubated on ice for 30 minutes, and protein-containing supernatants were collected by centrifugation at 4°C for 30 minutes. Bicinchoninic acid assay (BCA Protein Assay Kit, Pierce) was used to quantify proteins. Lysates (20 µg of total protein) were run on 4% to 12% polyacrylamide gels (NuPAGE, Invitrogen) by SDS-PAGE, transferred to nitrocellulose membranes, blocked in TBS/0.1% Tween (TBST)/5% milk, and probed with antibodies against E-cadherin (Cell Signaling Technology, 3195S, 1:1,000 dilution, RRID: AB_2291471), vimentin (Cell Signaling Technology, 5741S, 1:1,000, RRID: AB_10695459), and β-actin (Sigma-Aldrich, A5441, 1:5,000, RRID: AB_476744). All primary antibodies were diluted in TBST/5% milk and incubated overnight at 4°C. Membranes were washed in TBST and probed with secondary antibodies anti-rabbit horseradish peroxidase (HRP; Dako, P0448, 1:2,000, RRID: AB_2617138) for E-cadherin and vimentin and anti-mouse HRP (Dako, P0447, 1:2,000, RRID: AB_2617137) for β-actin. All secondary antibodies were diluted in TBST/5% milk and incubated for 1 hour at room temperature. For BRCA1 Western blot, protein was lysed in RIPA buffer, run on 3% to 8% gel (NuPAGE, Invitrogen), and probed using BRCA1 (Santa Cruz, sc-6954, 1:200, RRID: AB_626761) and β-tubulin (Cell Signaling Technology, 2146S, 1:1000, RRID: AB_2210545) primary antibodies and anti-mouse HRP (Dako, P0447, 1:2,000, RRID: AB_2617137) and anti-rabbit HRP (Dako, P0448, 1:2,000, RRID: AB_2617138) secondary antibodies. Enhanced chemiluminescence was performed by incubating membranes with Tris-HCl (100 mmol/L), luminol (1.25 mmol/L), P. coumaric acid (0.2 mmol/L), and hydrogenic peroxide (0.009%) diluted in distilled water for 1 minute at room temperature. Membranes were developed by exposing X-ray films to emitted light. Cell lines used as controls were originally obtained as described in ref. 27. These were authenticated by short tandem repeat testing and tested for *Mycoplasma* routinely and prior to experimentation.

### IHC

IHC was performed as described in (19). Briefly, tissue samples from PDTXs were fixed in 10% neutral buffered formalin, embedded in paraffin, and subsequently used to extract 0.6-mm cores for tissue microarray construction. Tissue microarray IHC staining was performed on 3-μm-thick sections that had been deparaffinized and rehydrated on the automated Leica ST5020 system. Appropriate antigen retrieval treatment was performed, followed by antibody staining using Polymer Refine Detection System (Leica, DS9800) using the automated Bond-III platform. Heat-induced antigen retrieval (sodium citrate or Tris-EDTA) was performed at 100°C. Enzymatic antigen retrieval utilized Bond Enzyme Concentrate (Leica, AR9551; 101.8 μg/mL proteolytic enzyme concentration) at 37°C. The signal was enhanced using DAB Enhancer (Leica Biosystems) for all antibodies apart from estrogen receptor (ER) and progesterone receptor (PR). After staining, sections were dehydrated, cleared in xylene on the automated Leica ST5020 system, and mounted with the CV5030 system (Leica). HER2 staining was performed separately using the PATHWAY rabbit anti-HER2 mAb (Ventana, 790-2991) and iView DAB Detection Kit (Ventana, 760-091). All antibody details (including RRID) can be found in Supplementary Table S2.

### PDTX *ex vivo* drug screening

Cryopreserved PDTX tumors were dissociated into single-cell suspensions, as described above, and resuspended in cell culture media: RPMI-1640, supplemented with serum-free B27 (100×), EGF (20 ng/mL), FGFβ (20 ng/mL), penicillin–streptomycin (50 U/mL), and gentamicin (5 µg/mL). Cells were plated in 384-well plates using 50 µL per well at cell concentrations of 1 to 3 × 10^6^ cells/mL. Cells were cultured 24 hours prior to dosing. Cells were treated with the indicated compounds in triplicate using a seven-dose–response curve. All drugs except AZD1056 were dosed at 10, 3, 1, 0.3, 0.1, 0.03, and 0.01 µmol/L. AZD1056 was dosed at 1, 0.3, 0.1, 0.03, 0.01, 0.003, and 0.001 µmol/L due to solubility. Viability was measured by Cell-Titer-Glo-3D (CTG-3D; Promega, G9683) 14 days after dosing.

PDTX drug responses were analyzed as previously described in (19). Briefly, the observed response was computed as 100 − [100 × (intensity-negative control)/(positive control − negative control)]. Nonparametric isotonic regression using the *R* function isoreg was used to fit the set of technical replicates of a given drug response for a given sample. The AUC was computed on the model fits using the trapezoid rule with the R package flux.

### Data availability

All processed data generated from the PDTX models and processing code, including WES, bulk and single-cell RNA-seq (scRNA-seq), *in vivo* growth measurements, and *ex vivo* drug screening data, are publicly available in the GitHub repository at https://github.com/cclab-brca/Dynamics_TNBC_clinical_trial. The raw WES, bulk RNA-seq, and scRNA-seq data generated in this study are publicly available in the European Genome-Phenome Archive repository at https://ega-archive.org/studies/EGAS00001001913. All requests for the data deposited in the European Genome-Phenome Archive will be granted. All other raw data are available upon request from the corresponding authors.

## Results

### A cohort of TNBC PDTXs

**Figure 1.**
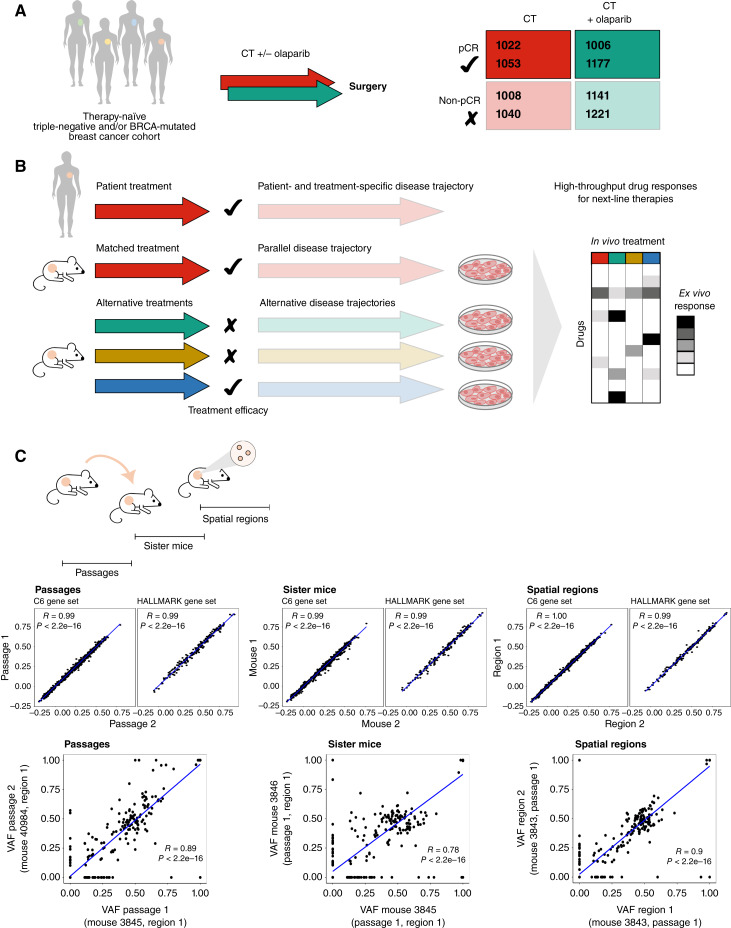
A preclinical platform of TNBC PDTXs. **A,** Clinical treatment and responses of the patient cohort from which the PDTX models used in this study were derived. **B,** Experimental framework of the coclinical trial. **C,** Top, correlation plots comparing GSEA enrichment scores (Hallmark and C6 gene sets) for models 1006, 1040, 1022, and 1141. Bottom, correlation plots comparing mutation VAFs for model 1006. Correlation was calculated between passages, sister mice, and multiple regions of the same tumor using Spearman correlation.

Patients with TNBC and/or germline *BRCA1/2* alterations were enrolled on a neoadjuvant clinical trial, PARTNER (ClinicalTrials.gov Identifier: NCT03150576; ref. 28), which tested the efficacy of the PARP inhibitor olaparib in combination with CT agents carboplatin and paclitaxel (Fig. 1A). Tumor samples at diagnosis (therapy-naïve) from consenting patients were collected fresh and immediately transported to the animal facility for s.c. engraftment into NSG mice, as previously described (Fig. 1B; ref. 19). A total of 67 independent samples were implanted, which resulted in 22 engrafted (grown as PDTXs) and 14 established models (successfully passaged). This 33% engraftment rate is comparable with that previously published by our group (19) and others (29).

PDTX models were subject to rigorous assessment by IHC and were shown to be of human epithelial cell origin, and breast cancer biomarker expression generally reflected that of the originating tumors (Supplementary Fig. S1; Supplementary Table S3). Of the 14 established PDTX models, 8 were used for the study described here; Supplementary Table S4 displays the clinical features. Most primary tumors were TNBC, and two tumors weakly expressed either PR or ER in the diagnostic samples. All tumors had a basal phenotype and were classified into integrative clusters 4ER-, 9, or 10. Three tumors had genomic *BRCA1* alterations, including a homozygous copy-number loss (model 1006) and a pathogenic mutation (c.4327C>T, p.Arg1443Ter) with loss of heterozygosity (model 1040).

RNA-seq from four models (1006, 1040, 1022, and 1141) was used to interrogate the impact of spatial heterogeneity on PDTX expansion by analyzing distinct spatial regions of the same tumor, sister mice within a passage, and across multiple passages (Fig. 1C; Supplementary Fig. S2). Principal component analysis and hierarchical clustering of gene expression data revealed clear separations between models, and samples from the same model clustered together. GSEA was performed on each sample, and we observed a high correlation between enrichment scores of both HALLMARK and oncogenic signature gene sets, C6, across spatial regions, sister mice, and passages within each model. To further explore this at the mutational level, we performed WES on spatial regions, sister mice, and across passages for one model (1006). This demonstrated a high correlation of the variant allele frequencies (VAF) of mutations between all levels.

This supported previous findings that PDTXs and associated experimental procedures were suitable for *in vivo* drug testing using sister mice as biological replicates and that the molecular features are stable across passages (19). This cohort presents an ideal experimental setting to assess parallel drug responses and explore genomic, phenotypic, and functional tumor evolution.

### A coclinical trial framework reveals concordant PDTX–patient drug responses

To test the concordance of drug responses between matched patients and PDTXs, we developed a coclinical trial platform. Crucially, these patient tumors and matched PDTX models were treatment-naïve and had deep molecular and clinical annotation, and the *in vivo* treatment regimen mirrored the clinical trial schedule.

**Figure 2.**
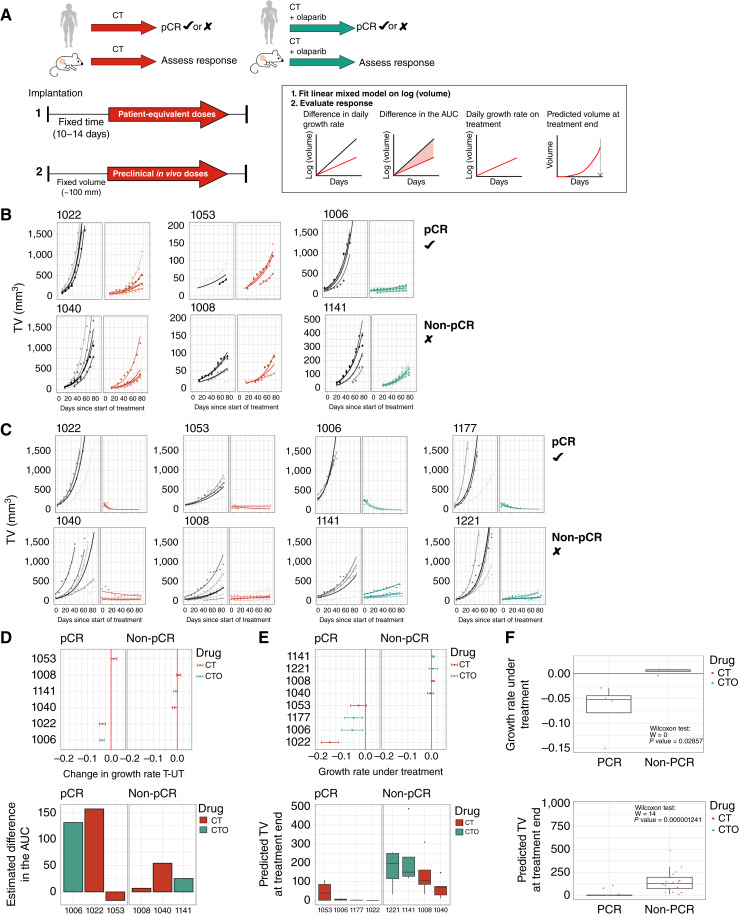
A coclinical trial framework reveals concordant patient–PDTX drug responses. **A,** Experimental framework (consisting of two trial designs) and associated analytical approach, with modeling metrics used to assess drug response. **B** and **C,** TV growth curves displaying linear mixed model fits of trial designs 1 (**B**) and 2 (**C**) over treatment duration. Treatment arm for each PDTX model corresponds to the clinical treatment of the matched patient. **D** and **E,** Analytical metrics derived from mathematical modeling (as in **A**). Change in growth rate (top) and estimated difference in the AUC (bottom) for trial design 1 (**D**) and growth rate under treatment (top) and predicted volume at treatment end (bottom) for trial design 2 (**E**). **F,** Box plots displaying growth rate under treatment (top) and predicted volume at treatment end (bottom) for trial design 2 between pCR and non-pCR models. Statistical significance is calculated using the Wilcoxon test.

We developed and tested two experimental trial designs and an analytical framework to evaluate response (Fig. 2A). For both, PDTX models were horizontally expanded into cohorts of daughter mice (mean of *n* = 5 per cohort following statistical power analysis). In design 1, to echo the PARTNER clinical trial regimen, treatment was administered 10 to 14 days after engraftment. In design 2, treatment commenced at a fixed TV (approximately 100 mm^3^) to account for model-specific growth dynamics; mice were assigned into cohorts using a stratified randomization approach, which aimed to evenly distribute initial TVs. For both trial designs, mice were administered with the same treatment as the matched patient (avatar mice), mirroring the patient treatment schedule and administration routes. In addition, the growth trajectory in the absence of treatment was monitored in five mice (untreated mice). Patients and PDTXs were treated with CT (carboplatin and paclitaxel) +/− olaparib, referred to as CTO and CT trial arms, respectively.

For design 1, mouse doses were chosen to be equivalent to the human doses in the clinical trial using relative body mass ratios (human 60 kg and mouse 20 g). The human dose of carboplatin (AUC5) was converted to 0.16 mg fixed dose for mice. The human paclitaxel dose of 80 mg/m^2^ was converted to 0.04 mg but, based on previous tolerability data on NSGs in our group, 0.07 mg was administered. The olaparib dose was converted from a 150 mg human dose to a 0.05 mg mouse dose. For design 2, drug doses were increased to 40, 7, and 50 mg/kg for carboplatin, paclitaxel, and olaparib, respectively, after performing tolerability experiments in NSG mice; approximate doses for a 20 g mouse were 0.8 mg carboplatin, 0.14 mg paclitaxel, and 1 mg olaparib.

For both trial designs and to mirror the clinical treatment schedule, carboplatin was administered once every 3 weeks by i.v. injection, paclitaxel was administered intravenously once weekly, and (when applicable) olaparib was administered by oral gavage on days 3 to 14 of each cycle. Four cycles of therapy were administered, with 3 weeks per cycle, totaling 11 weeks (77 days) of treatment. Although patients enrolled on the clinical trial received three subsequent cycles of anthracycline CT, this was omitted for the *in vivo* trial due to tolerability concerns for the mice.

Both trial designs were tested in six PDTX models spanning both clinical trial arms (CT and CTO), and trial design 2 was expanded to two further PDTX models. PDTX models were assigned into two response groups based on the matched patient’s clinical response. pCR and non-pCR were used to classify patients, which is a strong predictor of clinical relapse (30). The non-pCR patients used in this study had residual cancer burden (RCB) scores of RCB I or RCB II (Supplementary Table S4).

These data revealed high heterogeneity in growth rates between PDTX models, which was particularly pronounced using trial design 1 (Supplementary Fig. S3). This highlighted that the commencement of treatment at a fixed time point after implantation was not suitable for models with slower proliferation, e.g., 1008 and 1053. For these models, several untreated mice did not display measurable tumors after the treatment ended, and so drug responses could not be accurately assessed. In addition, for some models, we observed high variability between replicate mice in both untreated and treated cohorts; this was particularly noticeable for models 1040 and 1177. These data suggest that sample size should be adjusted for each PDTX model, guided by the untreated growth dynamics.

To account for this variability, we adopted mathematical modeling to assess the concordance with clinical drug responses (Fig. 2B and C). We used the assumption of exponential tumor growth (31) and fitted a single linear mixed-effects model for each unique PDTX model to estimate the drug response effect. The log-transformed TV was modeled as a function of time, treatment group, and their interaction. We included random intercepts and slopes for each mouse to model the variability in growth rates observed between replicates.

Four metrics were used to quantify the treatment effect: mean difference in daily growth rate; mean difference in the AUC; mean daily growth rate on treatment; and predicted TV at treatment end (Fig. 2D–F; Supplementary Fig. S4). The mean difference in daily growth rate and the AUC between the estimated marginal mean growth of the treated and untreated groups were used to assess whether treatment caused any improvement in the mice over untreated controls, even in instances in which the TV did not reduce. The mean daily growth rate for treated mice was used to identify whether treatment stopped tumor growth or induced tumor regression. The predicted volume at treatment end was the most similar metric to the pathologic assessment performed in the patient, measuring the total TV for each individual mouse at the end of treatment.

Using trial design 1, we observed marked differences in growth rate and the AUC between untreated and treated tumors for models 1022 and 1006, which were both derived from patients who achieved a clinical pCR (Fig. 2D). However, PDTX tumor growth was detected in all models. By contrast, using trial design 2, in which higher drug doses were administered, we observed improvements in treated mice compared with untreated controls in all models tested. However, tumor regression (indicated by negative growth rate under treatment) was observed only in models from patients who achieved pCR. Likewise, only models from these patients had a predicted TV close to zero at treatment end (Fig. 2E).

We used a Wilcoxon test to assess the statistical significance of each metric for the two trial designs. Trial design 2 produced statistically significant differences between pCR and non-pCR models using all four metrics (*P* < 0.05), whereas trial design 1 did not. As described above, trial design 1 was not suitable for models with slower growth rates (e.g., 1053 and 1008), whereas trial design 2 (commencing treatment at fixed TVs) accounted for these model-specific growth dynamics. Although we observed high variability between biological replicates for model 1053, the modeling data from trial design 2 revealed that the change in growth rate, growth rate under treatment, and TV at treatment end were all lower than all PDTX models from which the matched patient had non-pCR. Likewise, the difference in the AUC was higher in 1053 than all PDTX models from non-pCR patients. This demonstrates the applicability of our platform to all models tested, including those with highly variable growth dynamics.

These data comprise the first systematic assessment of concordant drug responses in a neoadjuvant treatment-naïve breast cancer setting using two experimental trial designs. Overall, we demonstrated an accurate recapitulation of clinical drug responses between matched patient–PDTX in eight models by adopting mathematical modeling to capture biological variability. Analytical metrics that capture improvements compared with controls were suitable for trial design 1, whereas trial design 2 was most appropriately assessed using growth rate under treatment and TV at treatment end. Analogous to clinical response measurements, predicted TV at treatment end produced highly significant results for trial design 2 when comparing pCR and non-pCR response groups (*P* = 1.241e−6), and we therefore propose that this is the most appropriate trial design and response metric to compare PDTX drug responses with the clinical scenario.

These data exemplify the platform’s utility in predicting patient drug responses, whilst concurrently highlighting crucial considerations for the design and implementation of preclinical trials, including intermodel heterogeneity and variability between replicate mice. These data set strong grounds for exploiting the use of PDTXs to test treatment strategies for matched patients.

### PDTXs identify patient-specific drug responses and model patient- and treatment-specific regrowth dynamics

**Figure 3.**
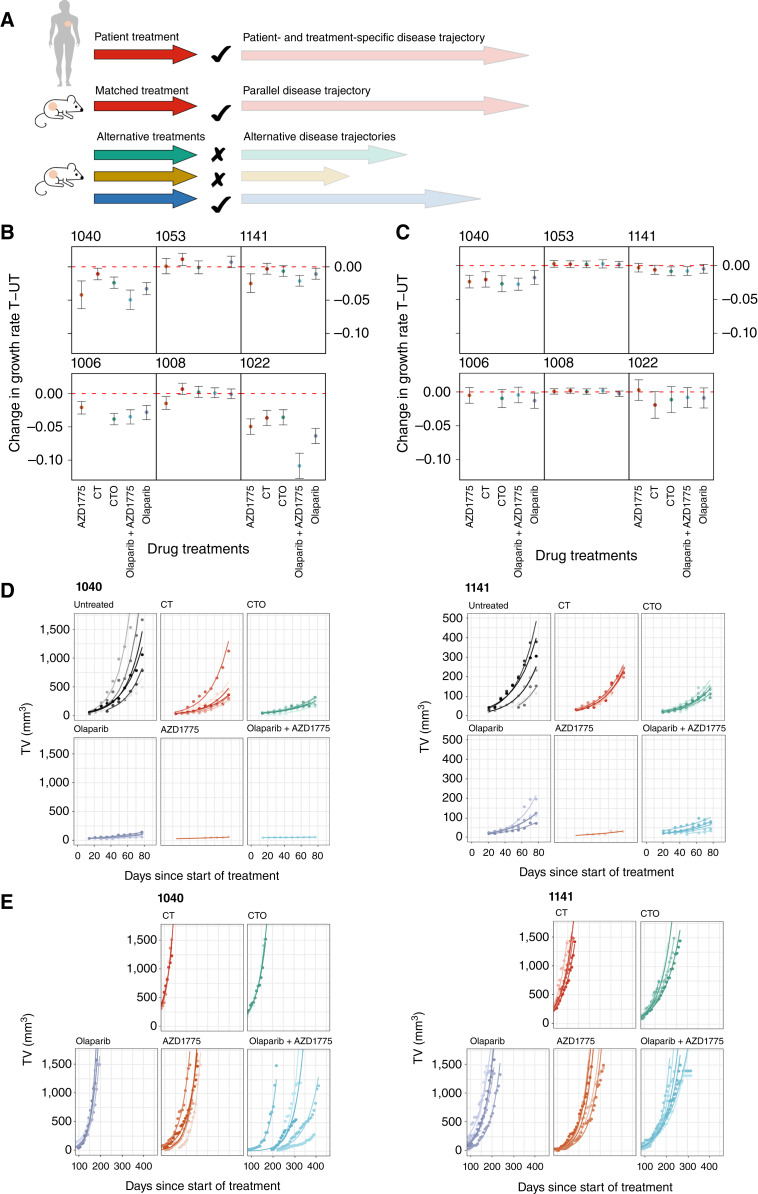
PDTXs identify patient-specific drug responses and model patient- and treatment-specific regrowth dynamics. **A,** Experimental framework to test drug responses to alternative treatments and regrowth dynamics. **B** and **C,** Mean difference in daily growth rate between the treated and untreated groups during treatment (**B**) and after treatment has ceased (**C**). **D** and **E**, TV growth curves displaying linear mixed model fits of PDTX models 1040 (left) and 1141 (right) during treatment (**D**) and after treatment has ceased (**E**). T–UT, treated–untreated.

We have demonstrated that PDTX models recapitulate clinical drug responses on a matched patient–PDTX basis. However, they also have potential utility as scalable tools in anticipatory cancer medicine to test alternative treatment strategies for patients and to explore patient- and drug-specific regrowth dynamics (Fig. 3A).

In this study, we expanded trial design 1 to include several other treatment arms. Using the same six PDTX models described in Fig. 2, cohorts of replicate mice (five mice per arm) were subject to 11 weeks of treatment with the same clinical trial arm to the matched patient, the alternative clinical trial arm ,and other exploratory therapeutic strategies. Olaparib was tested in the absence of CT, and AZD1775 (a WEE1 inhibitor) was also included (both as a monotherapy and in combination with olaparib). Patients with TNBC commonly present with DNA damage response (DDR) pathway impairment, and so AZD1775 was selected as an example of an alternative DDR-targeted treatment.

We implemented trial design 1 to aid in scalability; treatment commenced for all models 10 to 14 days after implantation. After treatment withdrawal, PDTX tumors were allowed to progress to size limits to model tumor regrowth. This enabled exploration of both drug efficacy on treatment and relapse dynamics for every patient–treatment pair. Replicate tumors were harvested at treatment end and at size limits for genomic and functional analyses.

We observed marked differences in drug efficacy and relapse dynamics in a patient- and treatment-specific manner (Supplementary Fig. S5). These data were analyzed using two different linear mixed models, during and following therapeutic pressure (Fig. 3B–E; Supplementary Figs. S6–S12), and response was reported using the same metrics as described above.

These results were particularly pronounced for models 1040, 1141, and 1022. For 1040, neither the patient nor the PDTX avatar responded to CT, whereas all alternative treatment strategies were successful at reducing growth rate during the treatment course (Fig. 3B; Supplementary Fig. S10). Once treatment ceased, all cohorts from model 1040 sustained a reduced growth rate compared with the untreated cohort, including CT, suggesting a prolonged drug effect (Fig. 3C). It should be noted that this in contrast to the other PDTX models tested, for which many treatment cohorts returned to comparable growth rates with untreated tumors upon drug withdrawal. We also observed striking differences in growth rate under treatment, TV at treatment end, and regrowth trajectories of model 1040 between treatment cohorts, exemplifying the platform’s utility to compare drug efficacy on a patient-specific basis. Also of note, we observed dramatic interreplicate heterogeneity in regrowth dynamics in the olaparib and AZD1775 combination cohort, which is captured using our modeling approach.

Model 1141 was largely resistant to treatments *in vivo* but responded to AZD1775 both as a monotherapy and in combination with olaparib (Fig. 3B; Supplementary Fig. S12). This was particularly evident by a change in growth rate and predicted volume at treatment end. However, upon drug withdrawal, the cohort treated with AZD1775 monotherapy returned to comparable rates to the untreated cohort, whereas the olaparib and AZD1775 combination cohort sustained a small reduction in growth rate (Fig. 3C). In stark contrast, model 1022 was highly sensitive to all compounds tested, but once treatment ceased, growth returned to comparable rates with the untreated cohort.

These data support the potential use of the PDTX platform as a scalable tool to test the effects of alternative treatment strategies on each patient’s tumor. However, it also illuminates two important findings. First, that testing on-treatment drug efficacy is insufficient to fully capture the long-term effects on a tumor. Second, first-line therapy can drastically alter the regrowth dynamics of a tumor, and these changes are patient-specific.

### PDTXs tumors undergo permanent nongenetic phenotypic evolution under therapeutic pressure

Having identified that the regrowth dynamics resulting from *in vivo* treatment are both patient- and treatment-specific, we reasoned that this may have important consequences for the use of PDTXs as preclinical models both in personalized medicine for matched patients and in preclinical trials. The effect of previous treatments (either in the patient or PDTX) on a tumor’s phenotype, evolution, and functional characteristics remains unexplored. Our platform provided the ideal setting to perform a detailed analysis of this question.

We chose to explore the genomic and phenotypic landscape of model 1006 when treated with the clinical trial compound, olaparib. Model 1006 was selected as a prototypical homologous recombination–deficient tumor with both germline and somatic *BRCA1* copy-number loss. As expected, model 1006 did not express BRCA1 at the RNA or protein level (Supplementary Fig. S13).

**Figure 4.**
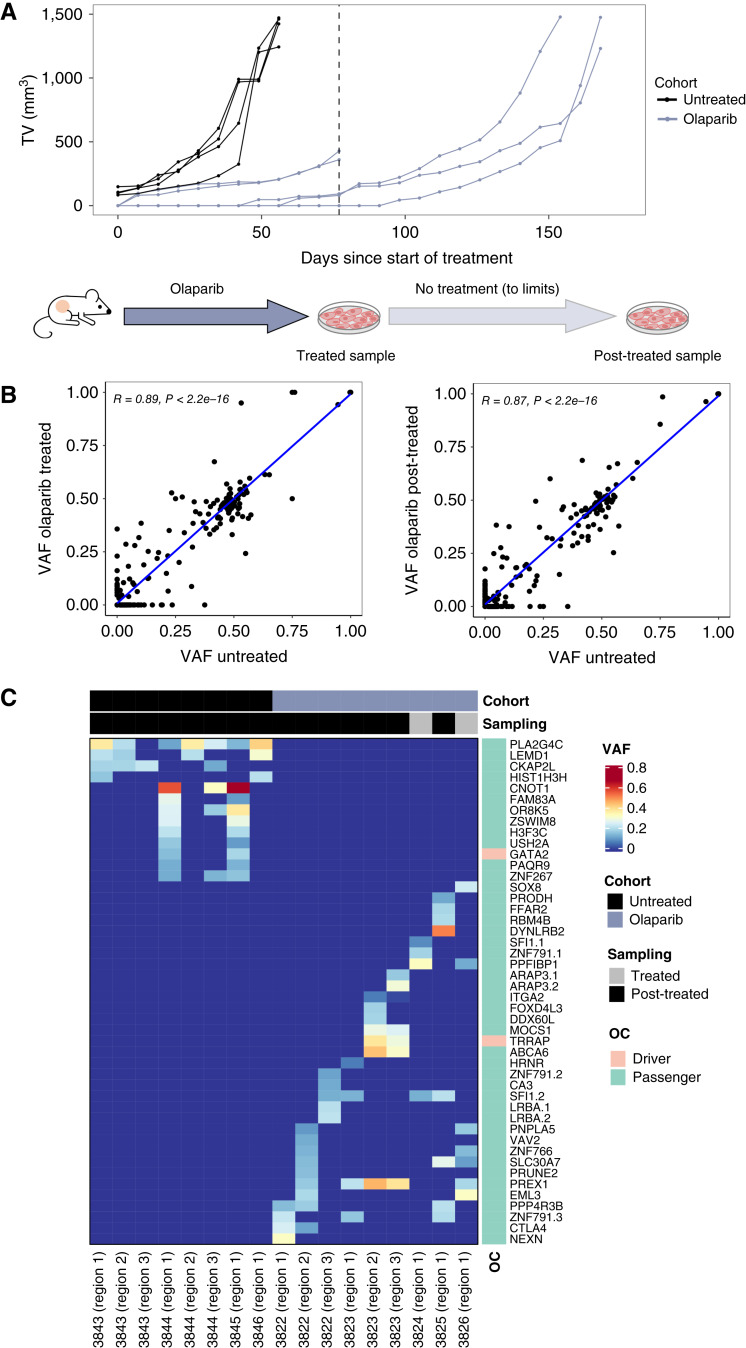
Short-term olaparib treatment does not cause major genomic changes. **A,** Growth curves displaying raw data of model 1006 treated with olaparib for 11 weeks (purple) or untreated controls (black). Dotted line indicates end of treatment, after which, tumors were left to progress until size limits. **B,** Correlation plots comparing mean VAFs of mutations between untreated and treated (left) or untreated and post-treated (right) samples. *R* value calculated using the Spearman correlation. **C,** Heatmap displaying VAFs of depleted and emergent mutations in each sample. Columns indicate samples (labeled by mouse number and intratumor region). OC, oncogenic classification as predicted by Cancer Genome Interpreter.

Model 1006 responded to olaparib treatment both as a monotherapy and in combination with CT (mirroring the patient response). However, intriguingly, after drug withdrawal, these tumors exhibited rapid regrowth, suggesting that there is a reservoir of drug-tolerant cells that can resume proliferation (Fig. 4A). To study these, samples were collected from replicate mice immediately after treatment (treated) and at size limits (post-treated) modeling residual and relapsed disease, respectively.

We first performed a deep molecular characterization of the tumors’ genomic landscape. PARP inhibitor resistance mechanisms rooted in genomics have been reported throughout the literature (32), and we reasoned that intratumor heterogeneity could act as a substrate for clonal evolution, enabling cells to regrow following therapeutic pressure. However, multiregion WES failed to identify major genomic changes upon treatment (Fig. 4B and C; Supplementary Fig. S14). We focused our analyses on nonsynonymous SNVs, frameshift indels, and stop-gain mutations and observed a high correlation between the VAFs detected in untreated, treated, and post-treated tumors. Hierarchical clustering of SNV VAFs revealed no clear separation between untreated, treated, and post-treated tumors. We then sought to identify “emergent” and “depleted” mutations. Emergent mutations were defined as those present in no untreated samples but in at least one treated or post-treated sample; conversely, depleted mutations were those present in two or more untreated mice but no treated or post-treated samples. Thieteen depleted mutations and thirty-three emergent mutations were detected. Although most were classified as predicted passenger mutations, we identified a depleted mutation in GATA2 and an emergent mutation in TRRAP, both predicted driver mutations (33, 34). However, because these drivers were detected in just two untreated mice and one post-treated mouse, respectively, they was deemed insufficient to attribute the observed relapse to natural selection of a preexisting genetic clone via Darwinian evolution. This emphasized that genomic analysis alone may not capture important ongoing changes within a tumor’s evolution.

Because genomic analysis failed to identify any mutations that could fully explain the survival of cells on treatment, we sought to explore the gene expression landscape. We analyzed untreated, treated, and post-treated tumors by bulk RNA-seq, which revealed minor yet significant changes in gene expression of known PARP inhibitor resistance markers related to DDR rewiring, including BRCA2, RAD51, and PALB2 (Supplementary Fig. S15A). This was despite an absence of mutations in these key DDR genes, further highlighting the importance of phenotypic (in addition to genomic) characterization.

Interestingly, when the same analysis was performed on model 1022, we observed a characteristic PARP inhibitor resistance mechanism. Model 1022 (transcriptomic BRCA1-null but no reported BRCA mutations) demonstrated a significant increase in BRCA1 expression in tumors treated with olaparib (Supplementary Fig. S15B and S15C). After treatment with olaparib, 5/5 mice demonstrated significantly higher gene expression, and indeed the most highly significant differentially expressed gene between untreated and post-treated tumors was *BRCA1* (logFC 9.06; *P* value 4.99 e−06), indicating an epigenetic reversion of gene suppression. These data demonstrate that our platform enables the identification of known single biomarkers of resistance to PARP inhibitors. It also further supports the importance of phenotypic characterization of models, even in the absence of genomic alterations.

**Figure 5.**
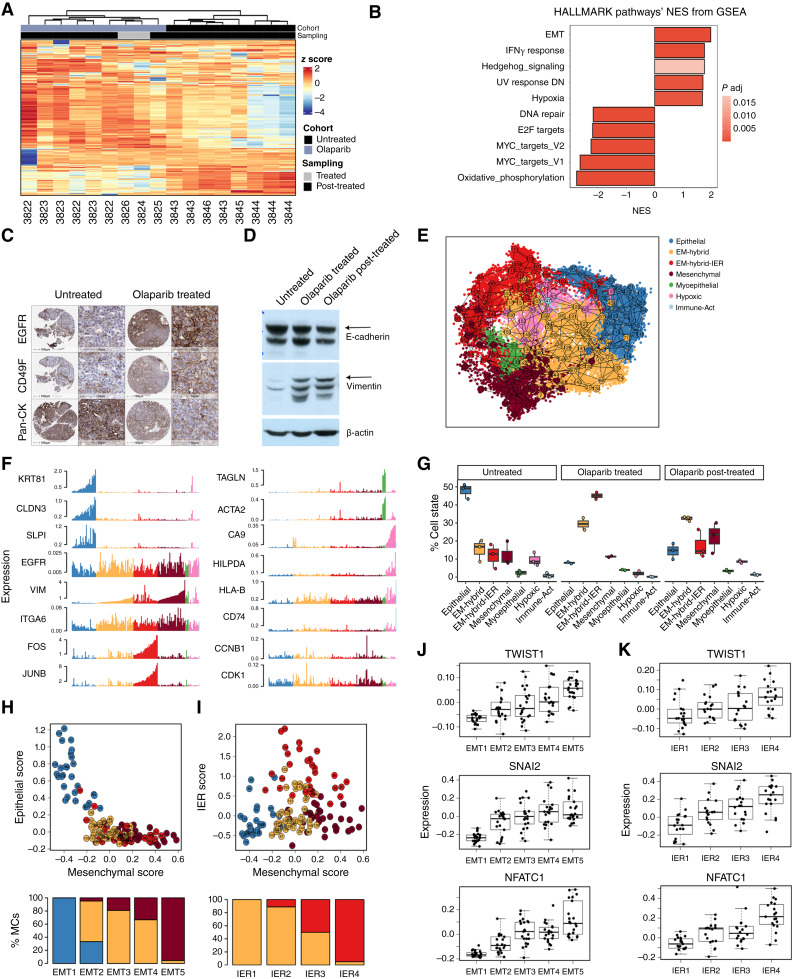
Olaparib treatment causes permanent phenotypic changes due to TF reprogramming. **A,** Heatmap displaying *z*-score (scaled by row) of the top 250 strong and variable genes. Clustering analysis performed using Euclidean distances. Columns indicate PDTX samples (labeled by mouse number). **B,** Top 10 significant gene sets by normalized enrichment score (NES) between untreated and post-treated samples, identified by GSEA (Hallmark gene sets). DN, down. **C,** IHC for phenotypic markers. **D,** Western blot for E-cadherin and vimentin. **E,** scRNA-seq data of all cells analyzed post-QC. Color indicates cell states (groups of MCs). **F,** Average gene expression (number of UMIs per 1,000 UMIs) of key markers across MCs. **G,** Percentage of cells from each condition that reside in each cell state. Immune-Act, immune activation. **H,** Top, epithelial and mesenchymal scores for individual MCs. Bottom, MC composition of each strata using mesenchymal minus epithelial scores to stratify MCs into 5 groups (EMT1–5). **I,** Top, mesenchymal and IER scores for individual MCs. Bottom, MC composition of each strata using the IER score to stratify MCs into 4 groups (IER1–4). **J** and **K,** Mean enrichment (log_2_ gene enrichment score) across strata for TFs of interest: EMT1–5 (**J**) and IER1–4 (**K**).

We then adopted an unbiased data-driven approach to explore the gene expression landscape of model 1006. Using the top 250 strong and variable genes identified in bulk RNA-seq, samples were clustered by Euclidean distance (Fig. 5A). Treated and post-treated tumors clustered together and separately from untreated tumors. To our surprise, this demonstrated a global transcriptomic shift upon treatment with olaparib that persisted after treatment was removed and ∼3 months of drug withdrawal. GSEA was performed between untreated and post-treated tumors (Fig. 5B; Supplementary Fig. S16), which demonstrated a significant enrichment of the HALLMARK “epithelial-to-mesenchymal transition (EMT)” gene set. Differentially expressed genes were then identified between untreated and post-treated tumors; post-treated tumors exhibited upregulation and downregulation of several mesenchymal and epithelial genes, respectively. These phenotypic changes were validated at the protein level (Fig. 5C and D).

To further interrogate the molecular mechanisms underlying these changes, scRNA-seq was used. A total of 21,894 cells from eight samples (at least two biological replicates per condition) were available for analysis after applying quality control filters. MC analysis (26) revealed seven groups of MCs (cell states; Fig. 5E and F; Supplementary Fig. S17). Minor cell states expressed high levels of myoepithelial (e.g., TAGLN and ACTA2), hypoxic (CA9 and HILPDA), and immune activation (HLA-B and CD74) markers. The remaining MCs were classified into cell states based on expression of epithelial (e.g., KRT81, CLDN3, and SLPI) and mesenchymal markers (e.g., VIM, ITGA6, and EGFR). MCs were divided into epithelial, mesenchymal, and EM-hybrid states, and a subset of EM-hybrid MCs expressed high levels of immediate early response (IER) genes (e.g., FOS and JUNB), which we denoted as EM-hybrid-IER.

Most cells in untreated samples exhibited an epithelial phenotype, consistent with bulk RNA-seq and protein data (Fig. 5G). Immediately after treatment, there was a dramatic decrease in epithelial and increases in EM-hybrid and EM-hybrid-IER cells. After treatment withdrawal, high levels of EM-hybrid cells remained (but fewer with IER expression), and an increase was observed in mesenchymal cells. We also observed a small increase in epithelial cells, but this remained dramatically lower than in untreated controls.

These data revealed two key phenotypic changes induced by olaparib treatment, with both transient and permanent dynamics. In line with our previous data, olaparib caused phenotypic changes involving sequential transitions from an epithelial to an EM-hybrid cell state on treatment and to a fixed mesenchymal cell state in relapse samples. Although we did observe a small degree of reversion to an epithelial phenotype, the overwhelming majority of post-treated cells remained EM-hybrid or mesenchymal. In addition, this scRNA-seq analysis revealed that olaparib induced transient high expression of IER genes in cells residing in a stressed cell state. Crucially, these changes were highly consistent between both biological and technical replicates.

To further elucidate the drivers of this, we calculated three gene expression scores (epithelial, mesenchymal, and IER) by averaging gene enrichment of the top 50 genes correlated with KRT81, VIM, and JUNB, respectively (Supplementary Fig. S18; Supplementary Table S5). Using expression data from epithelial, EM-hybrid, and mesenchymal MCs, each score was correlated with the expression of known transcription factors (TF; ref. 35). As expected, TFs that correlated with the epithelial score were negatively correlated with the mesenchymal score, and vice versa. Several TFs known to be associated with epithelial and mesenchymal phenotypes were indeed correlated with the respective scores, such as ELF5 (epithelial; ref. 36) and TWIST2 (mesenchymal; ref. 37). A subset of TFs was found to be correlated with both mesenchymal and IER expression scores, including EMT master regulator SNAI2 (38). This led us to hypothesize that the two key phenotypic changes observed in response to olaparib may be interrelated and that the transient overexpression of IER genes may drive, or be associated with, the permanent gene expression changes upon treatment.

Epithelial and mesenchymal scores were plotted for individual MCs, which (in line with several recent reports) demonstrated that EMT is a continuum rather than discrete cell states (Fig. 5H; refs. 39–42). Epithelial, mesenchymal, and EM-hybrid MCs were stratified into five groups (EMT1–5). Mean gene enrichment of TFs across the EMT1 to EMT5 strata was used to infer TFs that may be driving different stages of the EMT trajectory (Supplementary Fig. S19A–S19D). For some TFs, gene enrichment increased or decreased stepwise along the continuum, for example TWIST1 and ELF5, respectively. Others seemed early-acting, establishing a hybrid EM cell state, e.g., SNAI2 and ATF5. Conversely, other TFs were late-acting, transitioning cells from EM-hybrid to a fully mesenchymal phenotype, e.g., TWIST2 and IRX3. Adopting a similar approach for the IER trajectory (Fig. 5I), EM-hybrid and EM-hybrid-IER MCs were stratified into four groups (IER1–4). Again, mean gene enrichment of TFs was plotted across the IER1 to IER4 strata, which identified several TFs that correlated with acquisition of an IER phenotype, including TWIST1, SNAI2, and NFATC1 (Supplementary Fig. S19E and S19F). The observation that several TFs overlapped between the EMT and IER trajectories (Fig. 5J and K) supported our hypothesis that the transient and permanent phenotypic changes were interrelated. These data suggest that transcriptional programs associated with an IER trigger a cascade of phenotypic state dynamics, which occur independently or in parallel/interplaying with clonal evolution.

Overall, these data refine our understanding of tumor evolution in a setting that closely mimics the clinical scenario. These permanent changes driven by TF activity underscore a pivotal finding that even in the absence of new genomic alterations, there may be vast phenotypic changes affecting a tumor’s structure and function.

### *In vivo* treatment causes permanent changes to a tumor’s drug response profile

Having identified that *in vivo* treatment causes global phenotypic changes, which persist even after several months of drug withdrawal, we hypothesized that these changes may have functional implications. When considering drug responses, this could drastically impact the utility of PDTXs in the preclinical setting.

**Figure 6.**
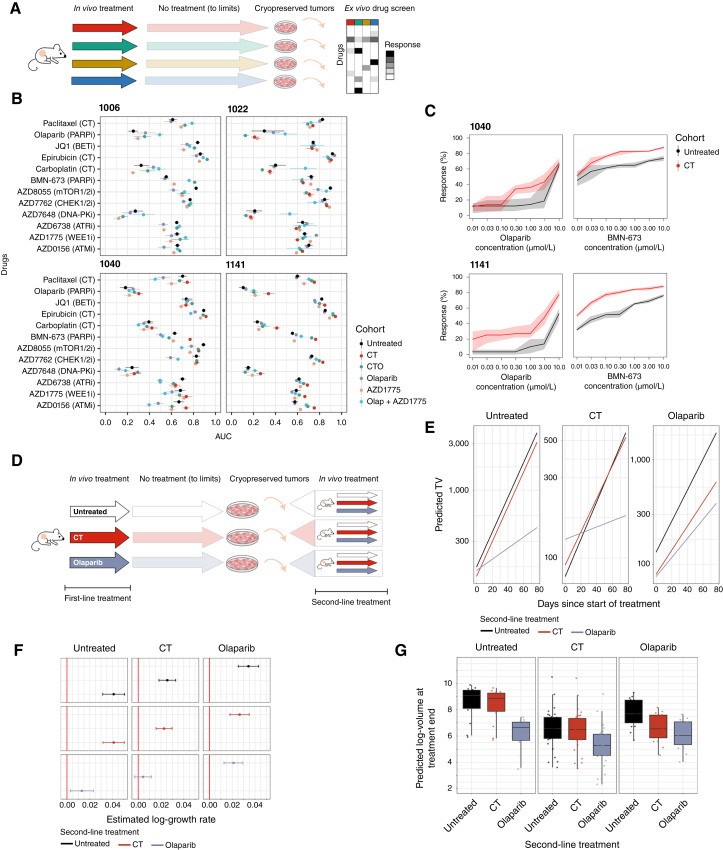
*In vivo* treatment causes permanent changes to a tumor’s drug response profile. **A,** Experimental framework of *in vivo* and *ex vivo* drug response profiling. **B,** High-throughput drug screening data of dissociated PDTX cells. AUC plots display the response of each tumor to drugs tested *ex vivo*. Color indicates the *in vivo* treatment to which each tumor was previously exposed. **C,** Dose–response curves of models 1040 (top) and 1141 (bottom) to olaparib and BMN-673. Plots compare the untreated PDTX tumors (black) to those previously treated *in vivo* with CT (red). **D,** Experimental framework to test alternative sequencing strategies of CT and olaparib *in vivo* using model 1040. Untreated PDTX tumors and those previously treated with CT or olaparib were each passaged into three cohorts of mice. These mice were left untreated or subsequently exposed to CT or olaparib. **E,** Estimated mean tumor growth for each experimental group. Panels display previous treatment groups (first-line treatment), and lines within each panel display the cohort of second-line treatment. **F,** Estimated log-daily growth rate of PDTX tumors, as predicted by the linear mixed models. Column panels display previous treatment groups (first-line treatment), and lines within each panel display the cohort of second-line treatment. **G,** Predicted tumor (log) volume at treatment end of each cohort treated sequentially with no treatment, CT, or olaparib based on the linear mixed models. Panels display previous treatment groups (first-line treatment), and boxes within each panel display the cohort of second-line treatment.

We performed a high-throughput drug screen on dissociated PDTX cells from post-treated samples using four PDTX models (1006, 1040, 1022, and 1141) and six *in vivo* cohorts (untreated and five treatments) described in Fig. 3 (Fig. 6A). We screened each sample with 12 drug compounds *ex vivo*. In total, we analyzed 276 *in vivo*/*ex vivo* combinations, providing a comprehensive understanding of the impact of *in vivo* first-line treatment on subsequent drug responses.

Our drug panel focused on DDR agents, which are relevant for the patient cohort who commonly present with DDR pathway impairment. This included PARP inhibitors (olaparib and BMN-673) and drugs targeting ATM, ATR, DNA-PK, WEE1, and CHEK1/2. A range of other drug classes were also included, such as CTs (paclitaxel, carboplatin, and epirubicin), epigenetic modulators (JQ1), and mTOR-inhibitors (AZD8055).

We observed significant functional changes, some of which were specific to both the patient and the *in vivo* first-line treatment to which each tumor was exposed (Fig. 6B). Shifts in the AUC between untreated and post-treated tumors indicate changes in sensitivity to the specific compound tested *ex vivo;* an increase in the AUC denotes a gain in sensitivity. For example, olaparib treatment *in vivo* decreased sensitivity of models 1040 and 1006 to several DDR agents (such as AZD6738, AZD7762, and AD1056), but the same pattern was not observed for models 1022 and 1141. It should also be noted that tumors were not always found to be less sensitive to the drug with which they were treated *in vivo* as a first-line therapy, likely due to the short treatment duration and the months of drug withdrawal.

We next analyzed these data for patterns across models and drug classes. This experimental framework has potential utility to identify efficacious and preferential sequential treatment regimens. We observed that *in vivo* treatment with CT increased response to the PARP inhibitors olaparib and BMN-673 for models 1040 and 1141 (Fig. 6C). However, the converse was not true; treatment with olaparib did not significantly affect *ex vivo* response to carboplatin for these models and decreased response to paclitaxel for 1040, indicated by a decreased shift in the AUC.

To validate these findings, we performed a preclinical trial in which we tested alternative drug sequencing strategies (Fig. 6D). Post-treated tumors collected from model 1040 after first-line *in vivo* treatment with either CT or ilaparib were each reimplanted into a cohort of 15 to 18 NSG mice. As controls, untreated tumors from 1040 were also reimplanted into 15 to 18 mice. When tumors reached ∼200 mm^3^, mice from each group were randomized into three trial arms and treated with no drug (untreated), CT, or olaparib (Supplementary Fig. S20A).

We observed high variability between mice within each cohort (Supplementary Fig. S20B and S20C), further exposing the crucial importance of both sample size and our linear mixed model approach, which captures this variability between mice. We used the same analytical approach as described previously and reported four modeling metrics to describe the drug responses of each previous treatment group (first line) to each subsequent treatment [(second line) Fig. 6E–G; Supplementary Fig S20D and S20E]. In line with our previous findings for model 1040, we observed low response to CT but good response to olaparib in tumors that were previously untreated. We also observed low response to CT in the groups previously treated with either CT or olaparib. However, strikingly, we observed an enhanced response to olaparib in tumors that had previously been treated with CT compared with olaparib as a first-line therapy. This finding was substantiated by the mathematical modeling metrics: this sequence of treatment (CT followed by olaparib) was the only scenario in which growth rate under treatment was reduced to 0 (Fig. 6F), and it produced the lowest predicted volume at treatment end than any other cohort (Fig. 6G). This validates our *ex vivo* drug responses and suggests that *in vivo* treatment with CT affects the subsequent response to olaparib for this PDTX model.

These data emphasize the value of combining *in vivo* and *ex vivo* drug screening to study PDTX drug responses and show that *ex vivo* drug response changes are recapitulated *in vivo*. This illuminates a previously unrecognized utility of PDTX experimental frameworks to identify efficacious sequential treatment strategies and to study the evolutionary, phenotypic, and functional consequences of treatment on the tumor.

## Discussion

PDTX models are excellent experimental tools to study human disease at high resolution, and the use of PDTX models in preclinical studies is widespread. Their utility ranges from molecular characterization of disease to identification of drug resistance mechanisms. Within drug development, PDTXs are exploited to screen experimental compounds, from early drug development to preclinical studies adopting 1 × 1 × 1 trial designs, in which cohorts of PDTXs mimic clinical trials (43, 44). More recently, PDTXs are gaining traction as predictive tools in personalized medicine.

We have built on our previously published findings to develop a coclinical trial experimental and analytical framework, anchored alongside a prospective clinical trial for patients with TNBC. We demonstrated that PDTX models accurately recapitulate clinical drug responses on a matched patient–PDTX basis. We also showed that *in vivo* treatment causes permanent phenotypic changes to the tumor, which is driven by TF activation, but is overlooked when using genomic analyzes alone. Furthermore, *in vivo* treatment has long-term functional implications for the tumor, including changing the drug response profile for next-line therapies.

Although we are not the first to report concordant patient–PDTX drug responses in breast cancer (11, 12, 45), our data highlight several important findings for the use of PDTX models in preclinical and clinical medicine. Our data display heterogeneity in growth rates of PDTX models both between models and (for some) between replicate mice. It is therefore imperative to consider the trial design, number of replicates, and analyses for each individual model. In this study, we present data using two trial designs, each of which had unique benefits and suitability for different PDTX growth dynamics and experimental requirements. To account for intermouse differences, we adopted mixed linear modeling that captures this biological variability and reported the findings using four metrics. We propose this analytical framework as an appropriate and necessary approach to analyze such data. However, our findings strongly oppose the use of 1 × 1 × 1 trial designs for some PDTX models, and careful consideration is crucial before adopting such methods.

We argue that our experimental approach has potential to accelerate both drug development studies and precision medicine. Whereas clinical trials require comparison to standard-of-care treatments, we have developed a framework that allows direct comparison of any given treatment to a true negative control (untreated) and that is scalable to test alternative treatments, schedules, and combinations in parallel. Crucially, this platform could have uses in the curative setting, in which it is notoriously difficult to test new drugs but has potential for strong clinical benefit.

We demonstrated that a tumor’s phenotype is permanently altered by short-term *in vivo* treatment even in the absence of genomic changes. This highlights that it is essential to evaluate the phenotypic concordance of matched patient–PDTX tumors, in additional to genomic analysis, to ensure the molecular and functional features are retained during PDTX establishment and propagation. In parallel, we observed that *in vivo* treatment had functional implications on a given tumor and that these effects are treatment specific. This suggests that the clinical history of a tumor may impact the accuracy of *in vivo* drug response profiling. All primary tumors used to derive PDTXs in this study were treatment-naïve, but this pivotal finding indicates that PDTX models may require parallel treatments to the matched patient to retain accurate predictive value. Further work utilizing longitudinal clinical samples is essential to resolve this.

The data described here illuminate a previously unrecognized utility of PDTXs to study efficacious sequential and combination therapeutic strategies. Our experimental framework can be used to test alternative drug sequencing to identify best practices for both treatment efficacy and within the context of long-term tumor evolution. We also demonstrate that therapies with similar on-treatment effects can cause marked differences in a tumor’s regrowth dynamics, justifying the use of PDTXs to study evolution.

Our data also alluded to a novel drug response mechanism to olaparib. This is akin to previous reports of phenotypic switching in response to treatment (46–52; bioRxiv 2023.12.07.570359). A striking observation was the permanent nature of the phenotypic change, contrasting with mechanisms such as drug-tolerant persister cells (53) but more in line with stable nongenetic drug resistance (48, 49). Our scRNA-seq data suggest that upon treatment, cells adopt an EM-hybrid cell state enriched for IER genes, but upon drug withdrawal, cells continue to progress toward a fully transitioned, stable mesenchymal phenotype. Previous reports have demonstrated that EM-hybrid cells exhibit higher metastatic potential (40) and tumorigenicity (54) than their epithelial or mesenchymal counterparts. Our data leads to the compelling hypothesis that drug withdrawal may drive cells further toward a mesenchymal cell state, which, although traditionally associated with a more aggressive phenotype than epithelial cells, has less metastatic potential and tumorigenicity than hybrid cells that emerge on treatment. Using the scRNA-seq data to derive EMT and IER trajectories, we identified that overlapping TFs (including EMT master regulator SNAI2) may be implicated in directing cell state transitions, highlighting the potential to identify plasticity-driver vulnerabilities (55). Crucially, we have also shown that phenotypic adaptation has functional implications that could impact responses to next-line therapies.

We acknowledge several limitations of our study. Patients in the clinical trial received neoadjuvant anthracycline therapy following the clinical trial compounds, which was omitted from the *in vivo* trial due to tolerability concerns. Whereas this could limit the accuracy of the matched patient-PDTX drug response data, our findings revealed striking concordance. We argue that this work provides a strong foundation and preliminary experimental framework to expand into larger patient cohorts and wider ranges of clinical compounds and clinical scenarios. In addition, the use of both PARP and WEE1 inhibitors used in this study are not currently in standard-of-care clinical practice. Although this limits the direct implementation of our findings, we again argue that the platform provides a foundation to expand into other drug treatments and patient cohorts. Ultimately, a clinical trial with an avatar arm is needed to consolidate the use of these data to benefit patients with cancer.

Second, NSG mice used in our study are severely immunocompromised. Although this was necessary for the propagation of PDTX models, we acknowledge the significant role of the immune system (and more broadly the tumor microenvironment) in drug responses and tumor evolution, particularly considering recent advances in the use of immune checkpoint inhibitors. Despite this, we observed strong concordance to patient drug responses, and further experiments are required to delineate the impact of the remaining mouse and human immune compartments on PDTX growth, response to treatment, and relapse dynamics. As such, we propose that our platform could provide a useful setting to test a range of drug treatments (including targeted agents) but limited to those not requiring immune modulation.

In addition, we recognize the low engraftment rate (∼33% of all implanted). Although this is in line with that previously published by us (19) and others (29), it presents a possible caveat that we are selecting a subpopulation of patients with more aggressive tumors. We acknowledge that we are unable to comment on the behaviors and patient–PDTX concordance of the unengrafted PDTX population, and this remains unexplored. Indeed, this highlights another important consideration when using PDTXs for personalized medicine; low engraftment rates restrict the use of PDTXs for clinical practice and we are, to date, limited in our ability to predict which tumors will successfully engraft.

In this study, we present a robust and systematic assessment of PDTX models as preclinical tools. We have developed a novel experimental and analytical framework to test patient–PDTX drug response concordance, assess drug efficacy, and explore the impact of alternative treatments on long-term evolutionary trajectories of human breast cancer. In doing so, we have illuminated new opportunities and considerations for the use of PDTXs in both preclinical and clinical practice.

## Supplementary Material

Supplementary Figures 1-20 Supplementary Figures 1-20

Supplementary Tables 1-5 Supplementary Tables 1-5

Supplementary Legends Supplementary Figures 1-20 and Supplementary Tables 1-10 Legends
