# Supplementary material for: Modeling Drug Responses and Evolutionary Dynamics Using Patient-Derived Xenografts Reveals Precision Medicine Strategies for Triple-Negative Breast Cancer
Source: Cancer Res. 2024 Nov 8;85(3):567–84. doi: 10.1158/0008-5472.CAN-24-1703 (PMC7617242; doi:10.1158/0008-5472.CAN-24-1703)
Supplement: Supplementary Figures 1-20 [file can-24-1703_supplementary_figures_1-20_suppsf1-20.pdf]

A

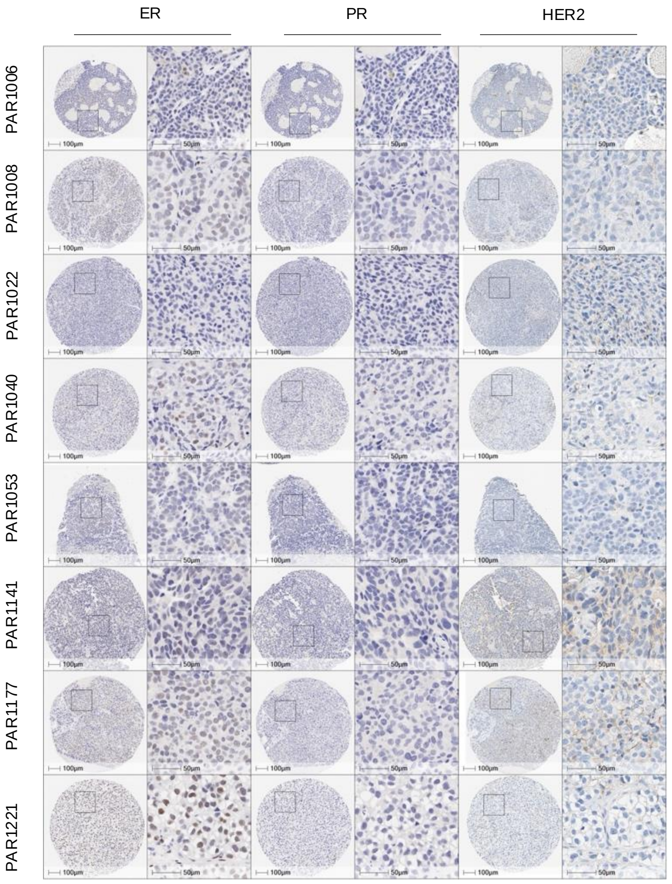

B

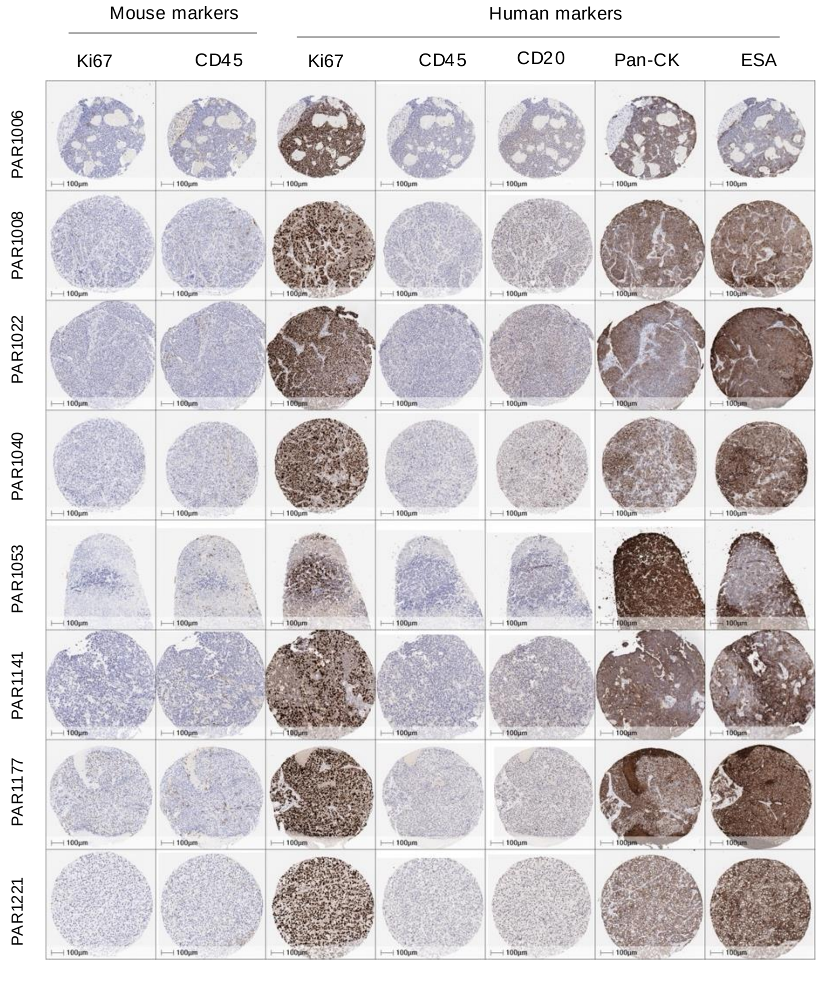

Supplementary Figure 1

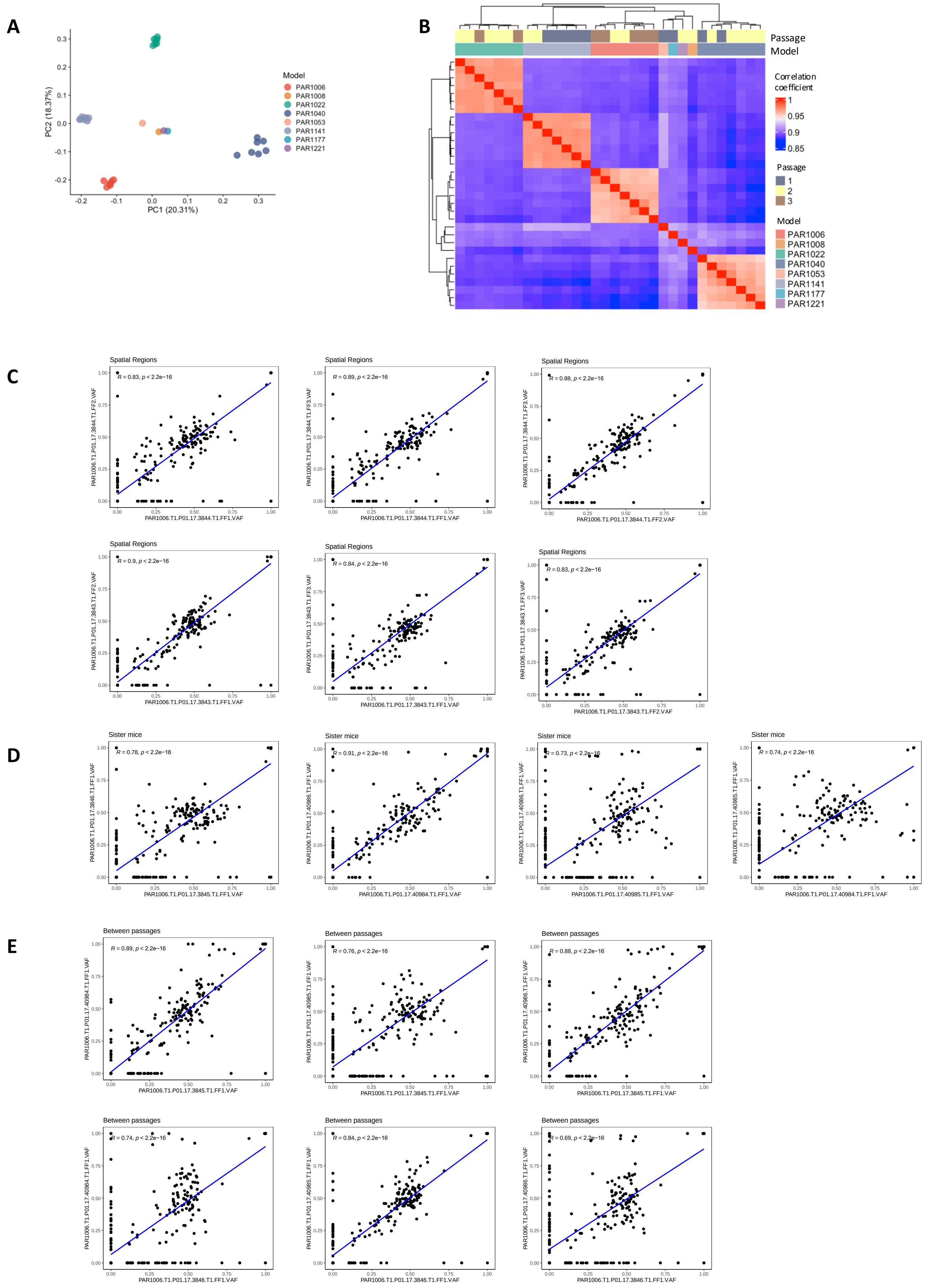

Supplementary Figure 2

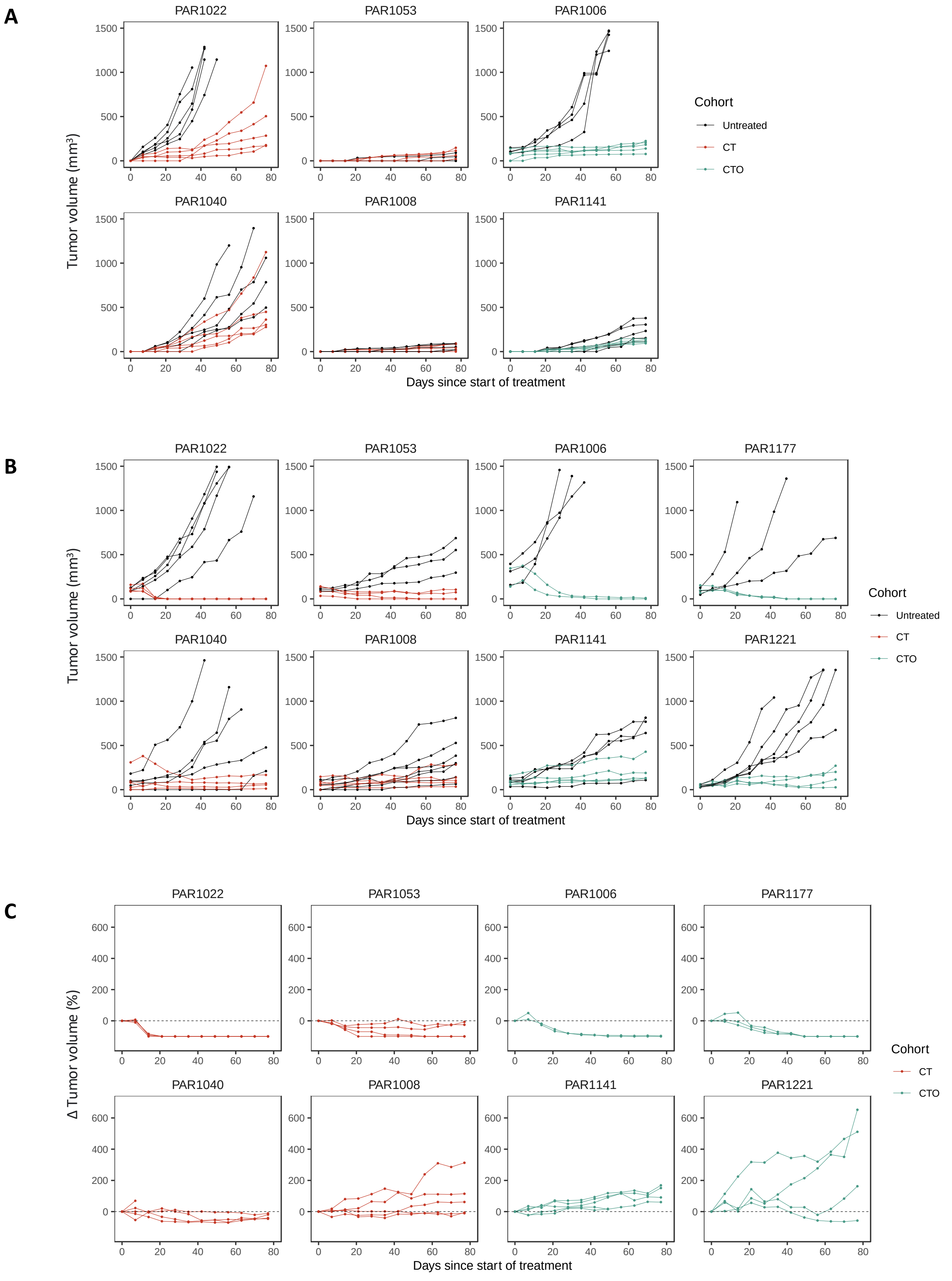

Supplementary Figure 3

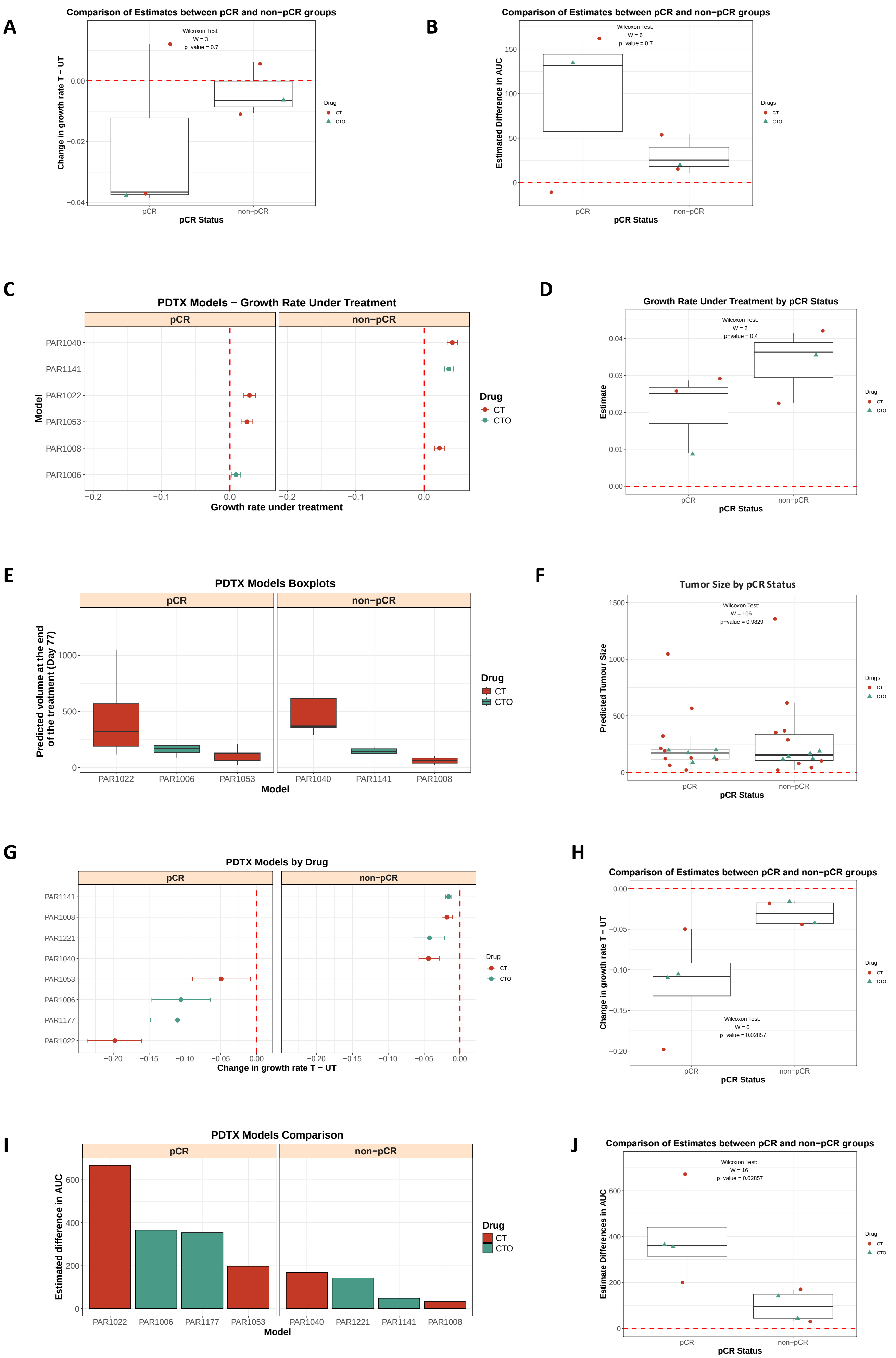

Supplementary Figure 4

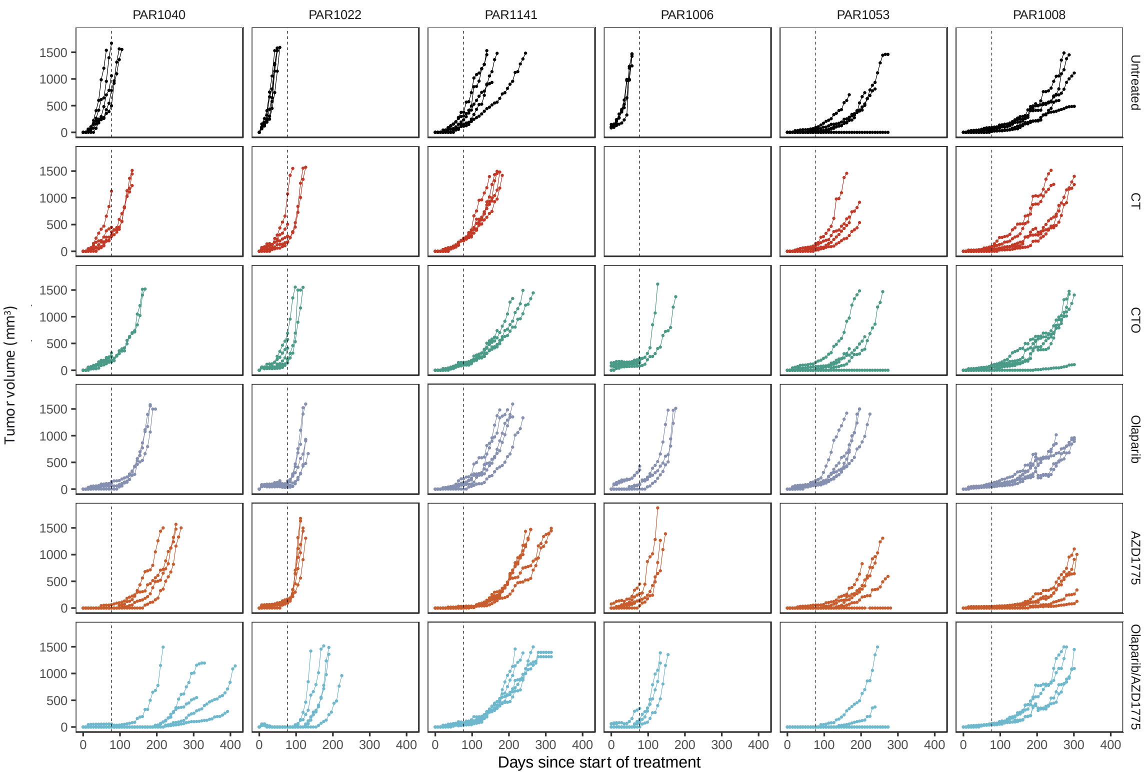

Supplementary Figure 5

A

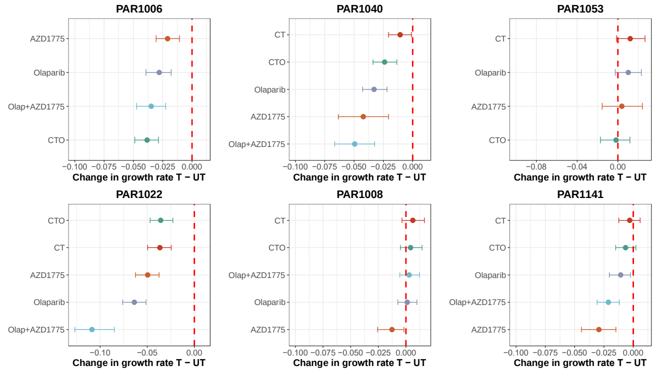

B

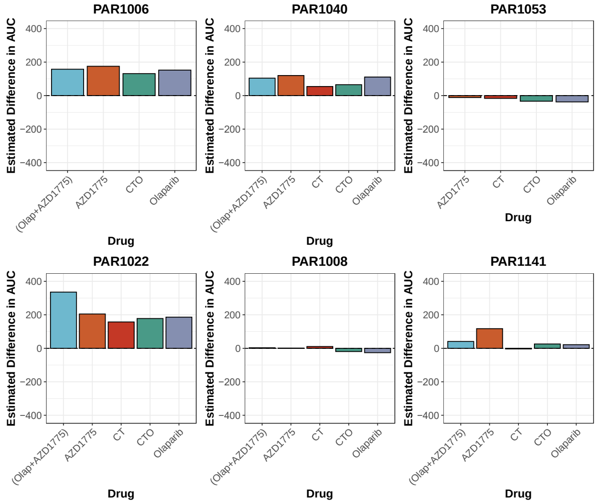

C

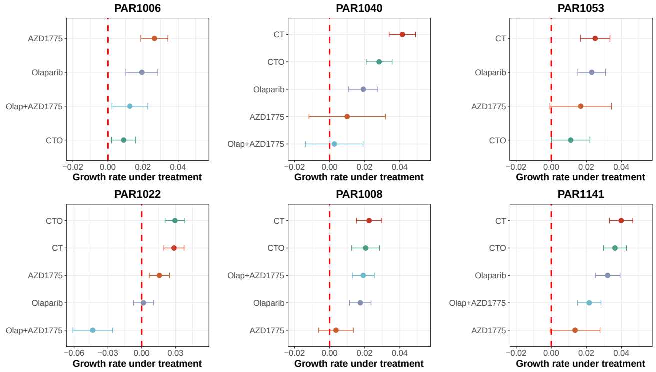

D

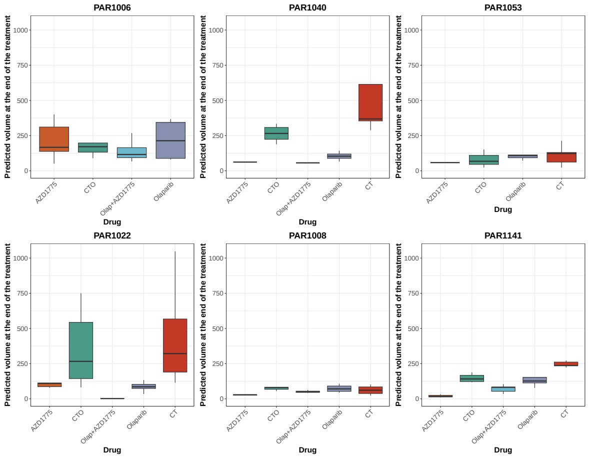

Supplementary Figure 6

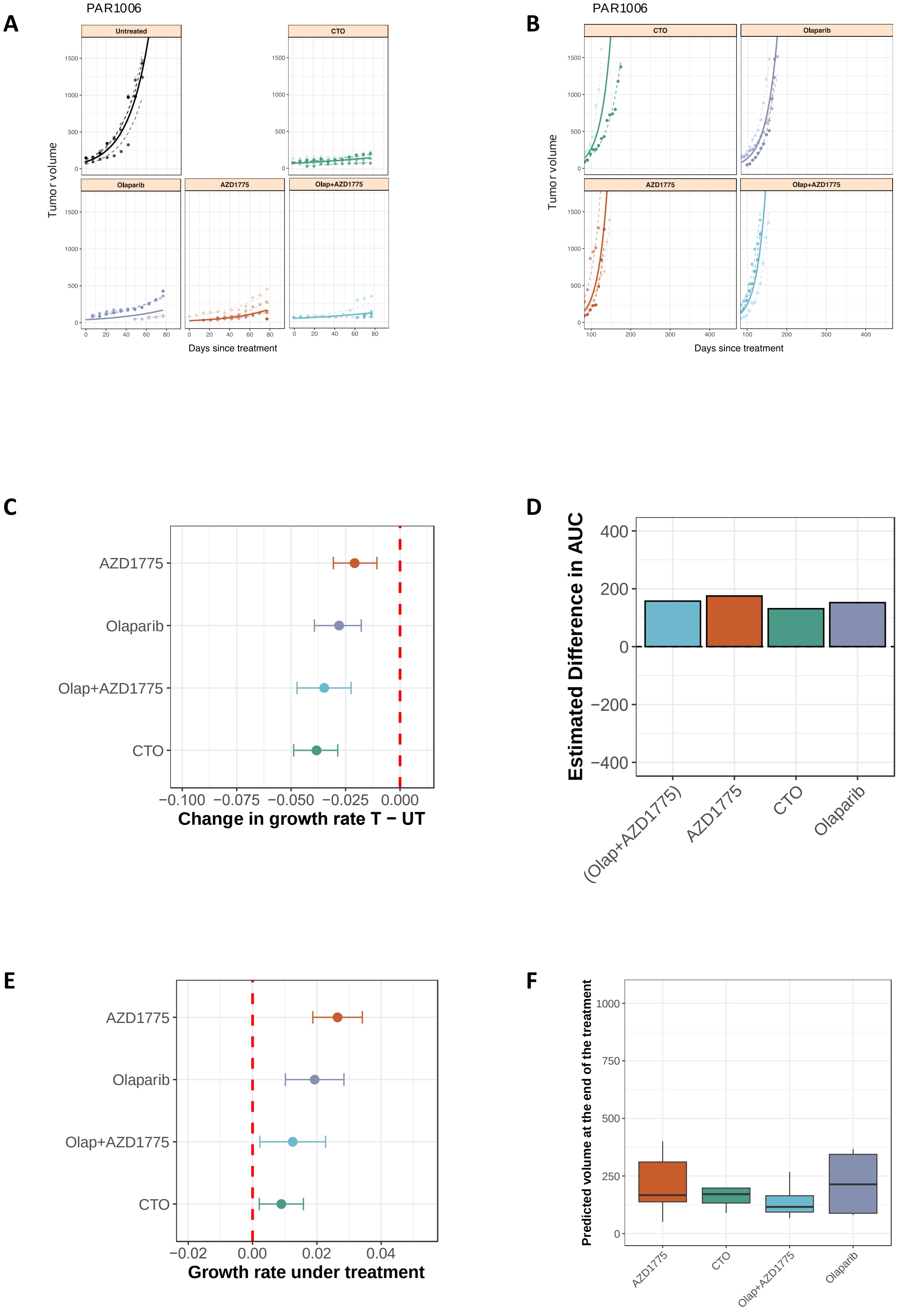

Supplementary Figure 7

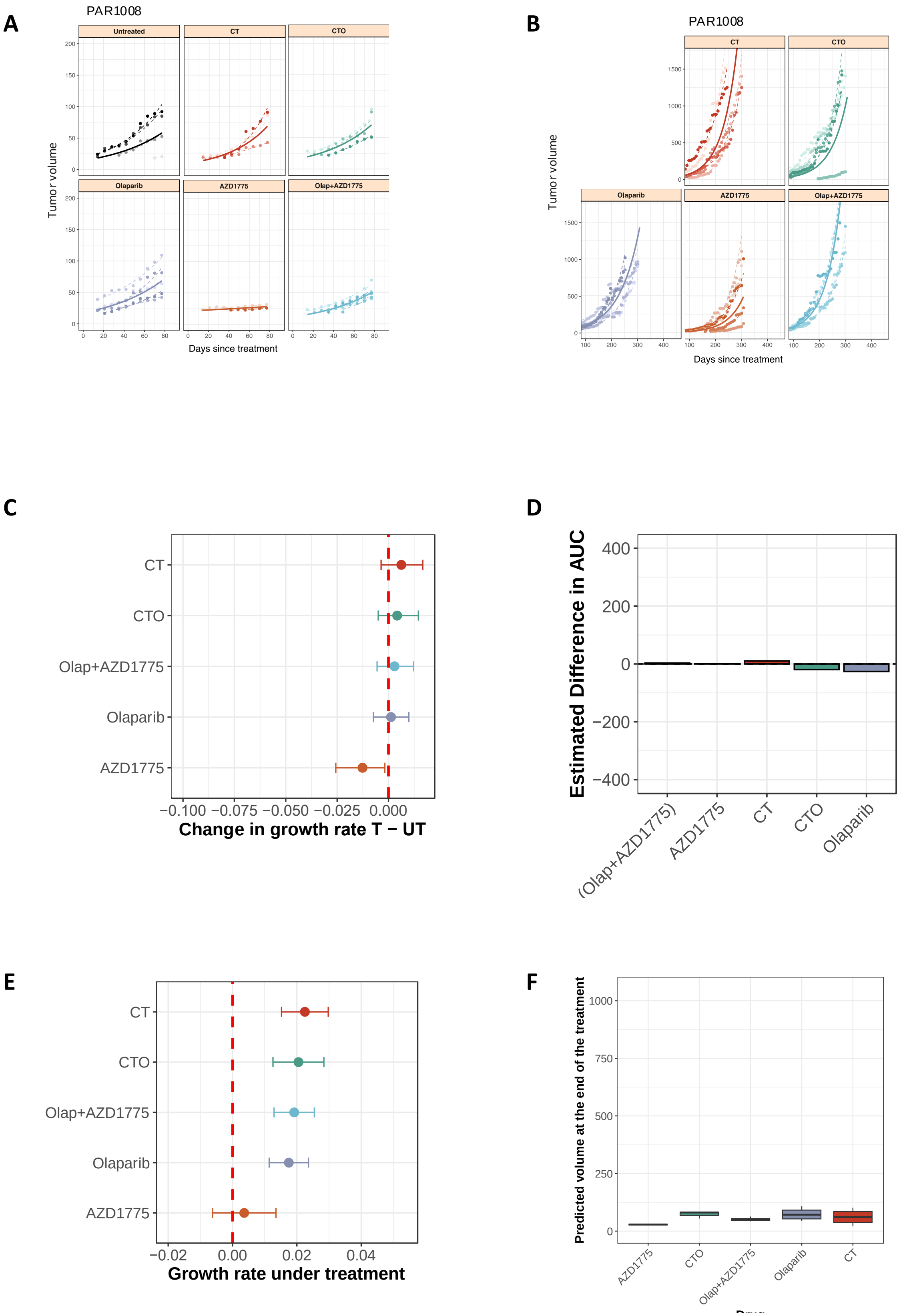

Supplementary Figure 8

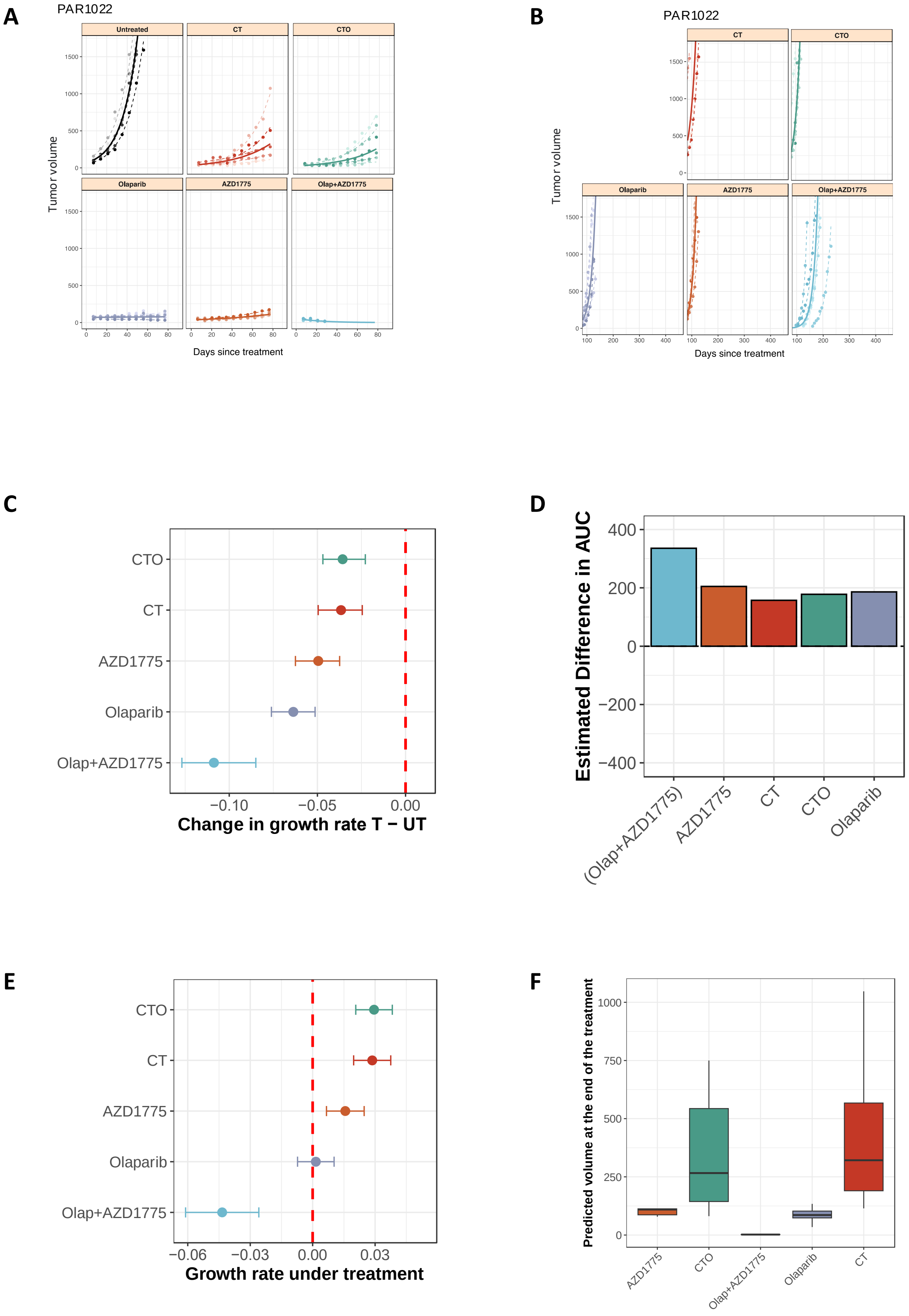

Supplementary Figure 9

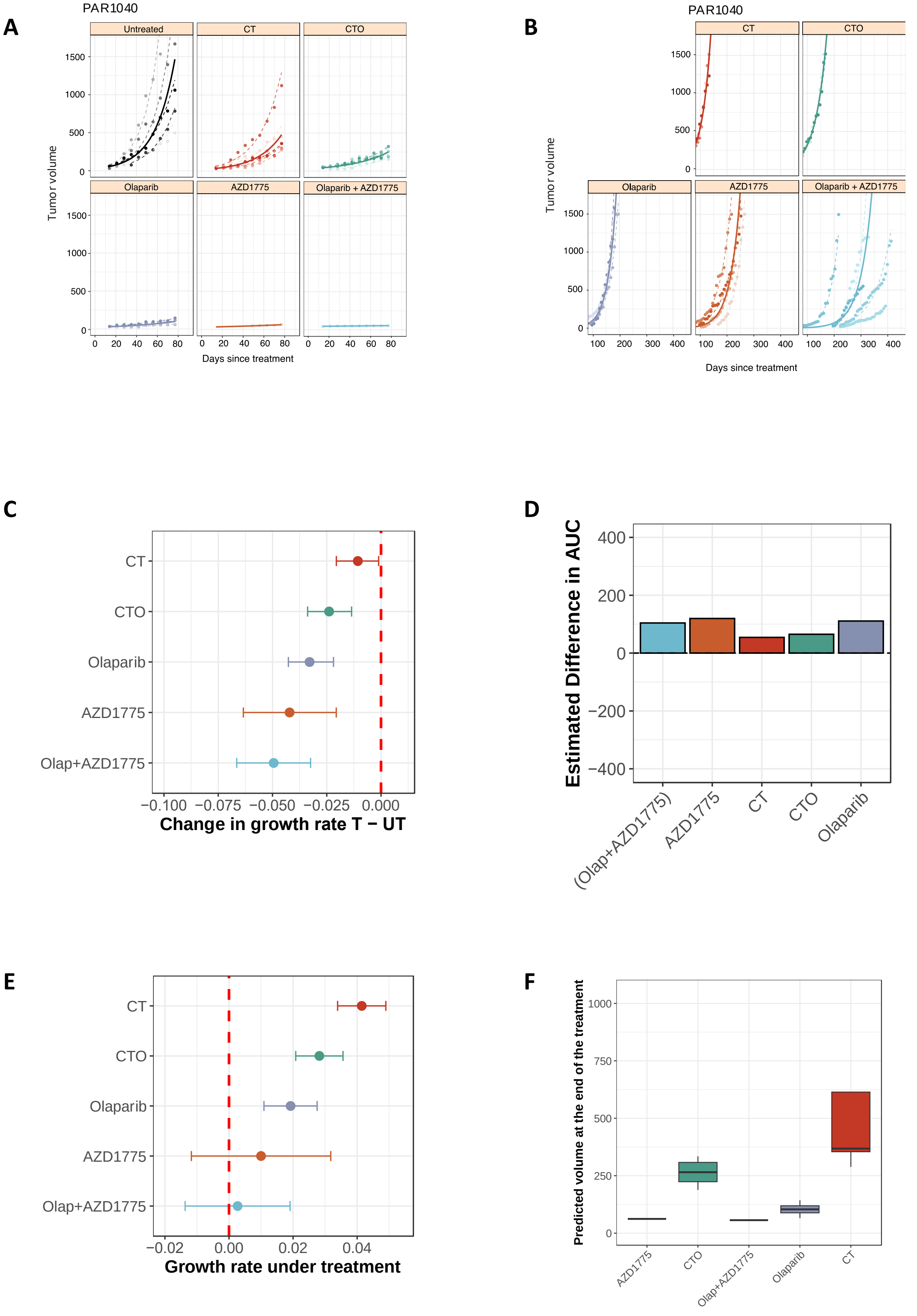

Supplementary Figure 10

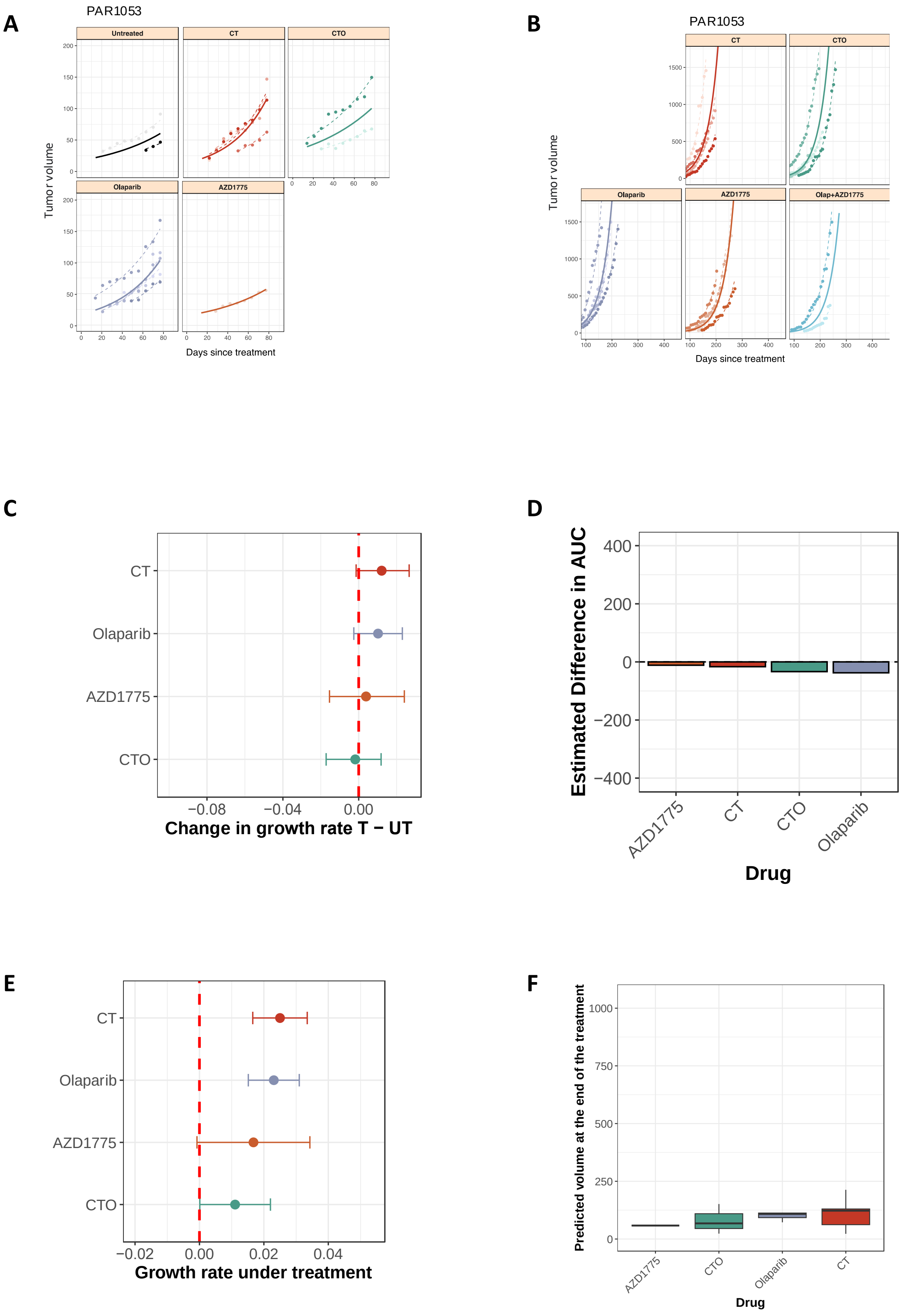

Supplementary Figure 11

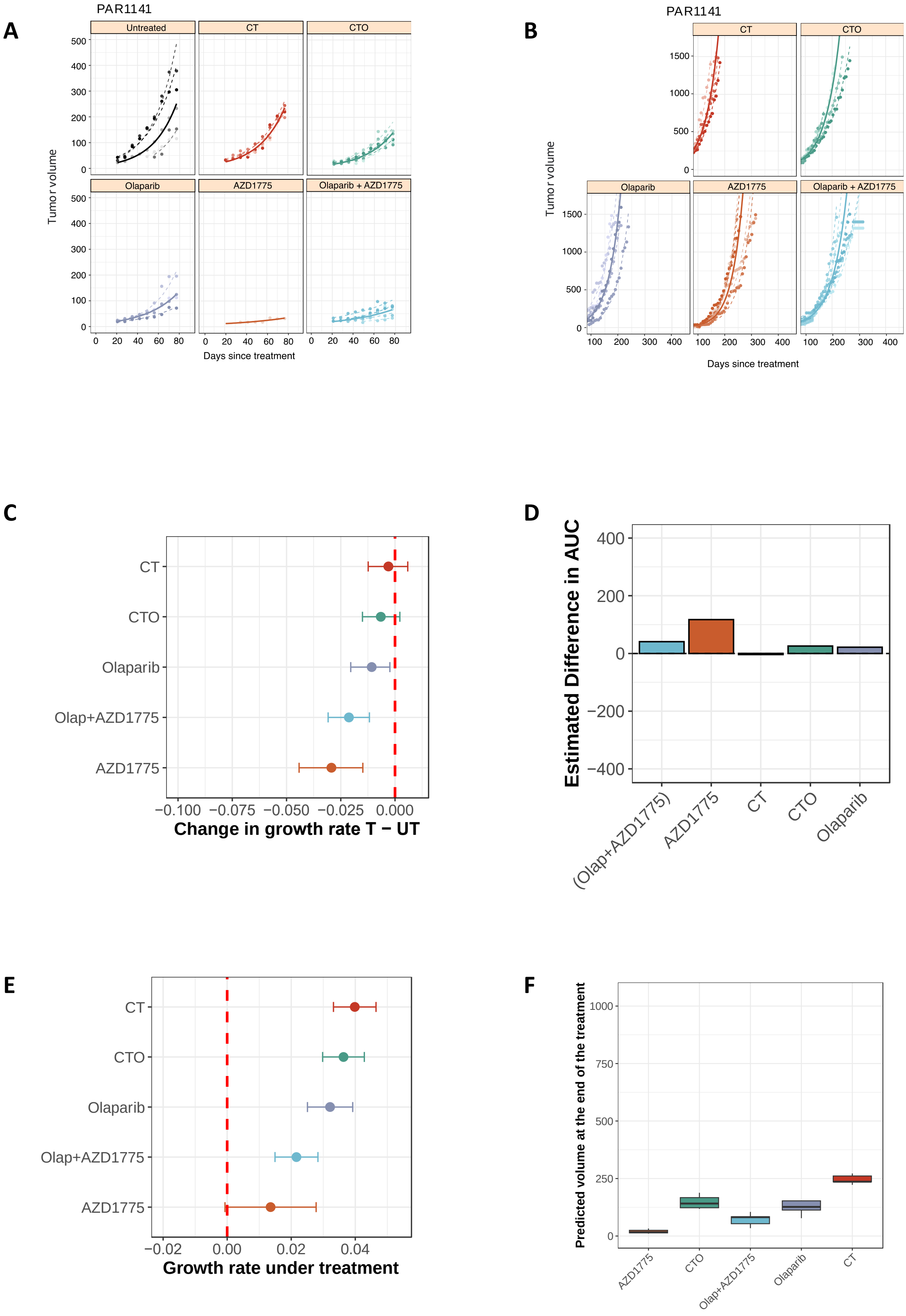

Supplementary Figure 12

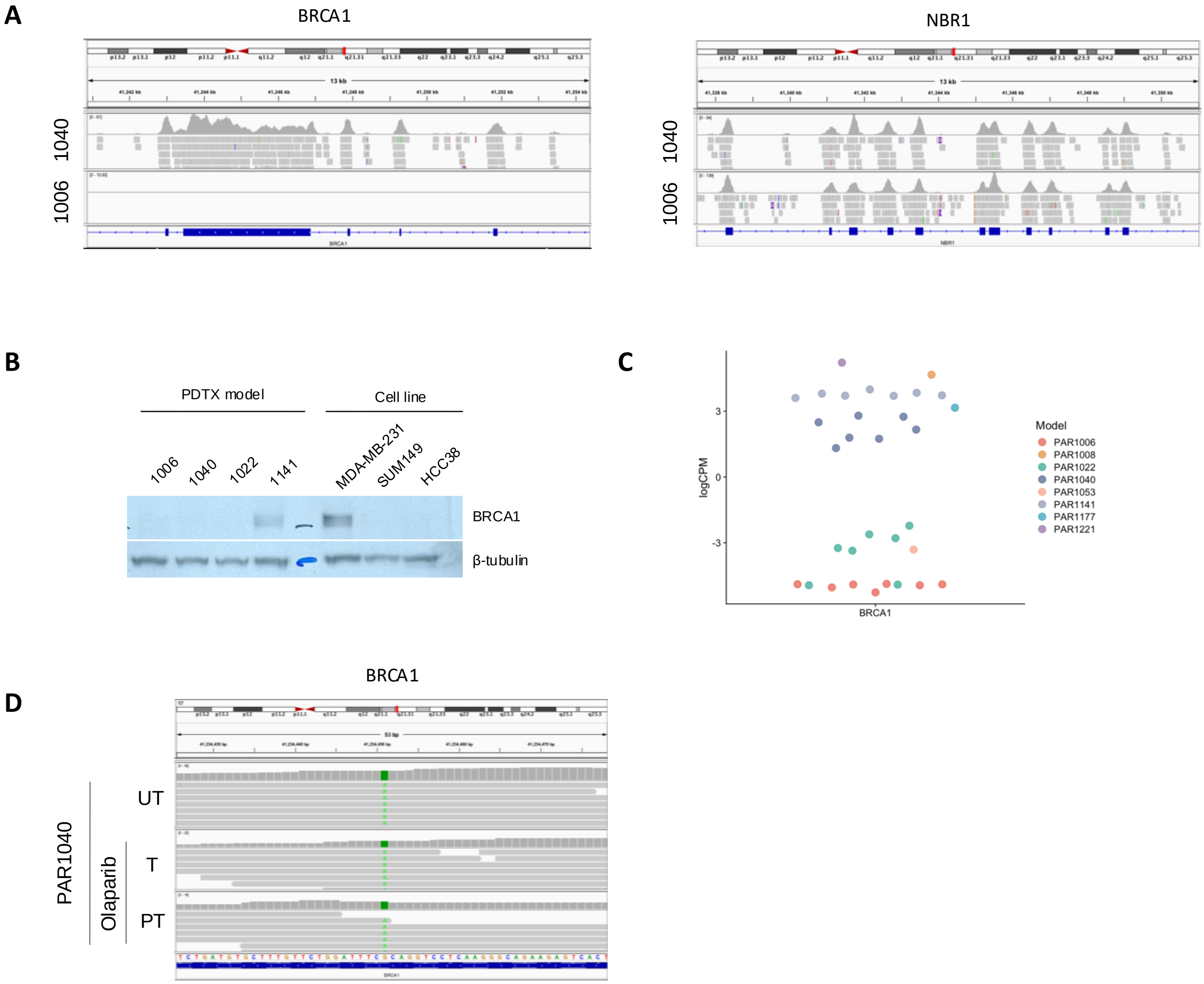

Supplementary Figure 13

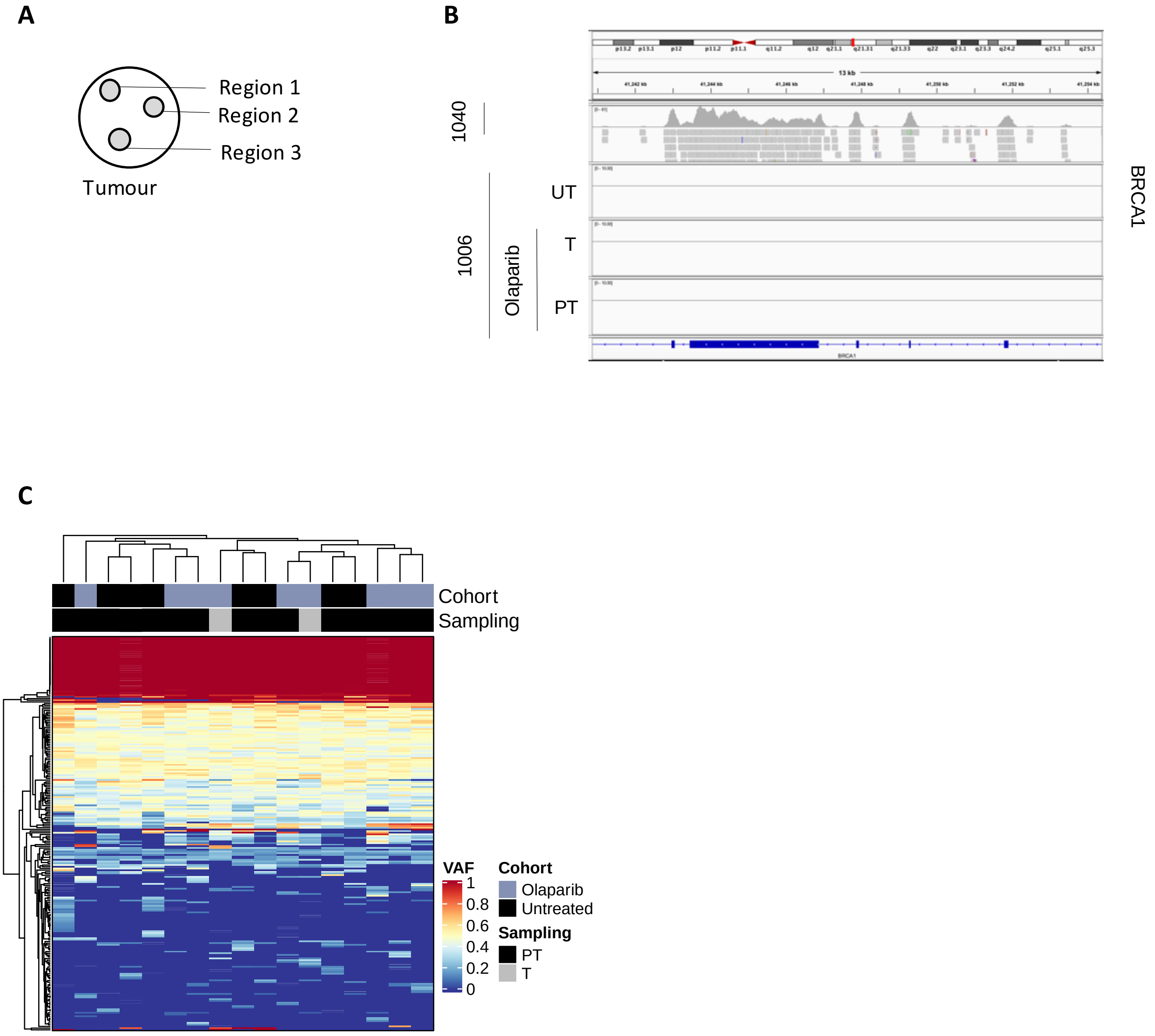

Supplementary Figure 14

A

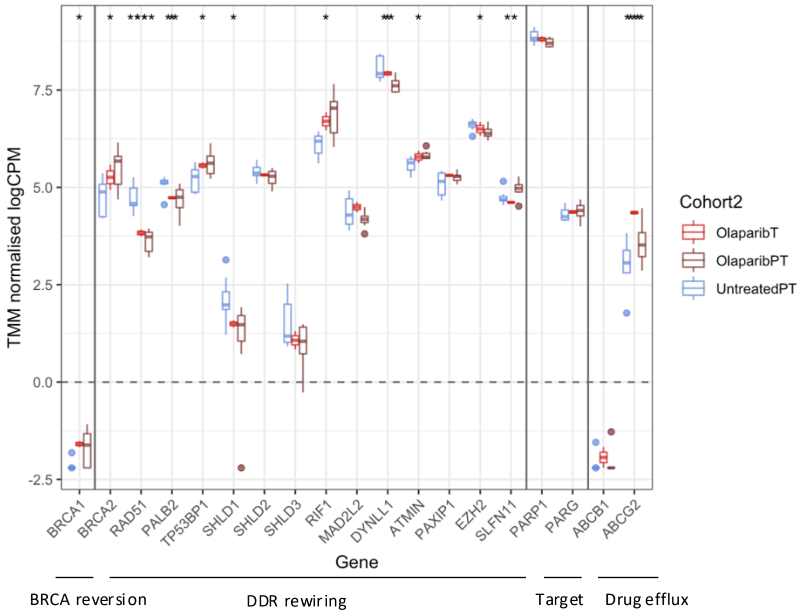

B

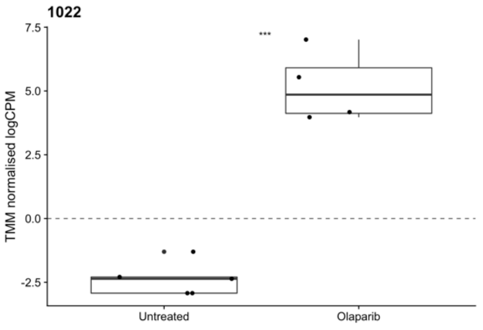

C

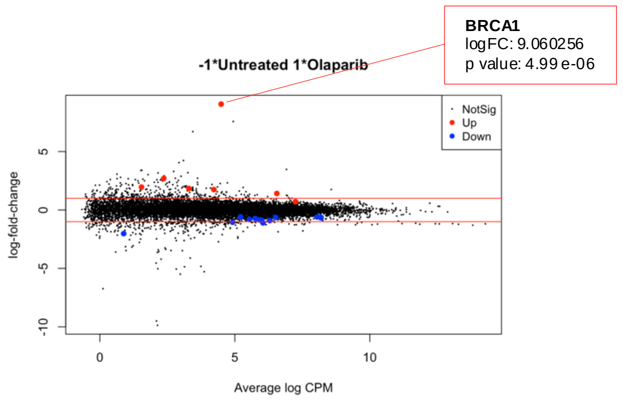

Supplementary Figure 15

A

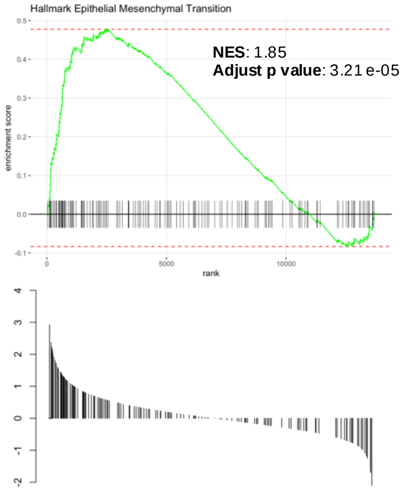

B

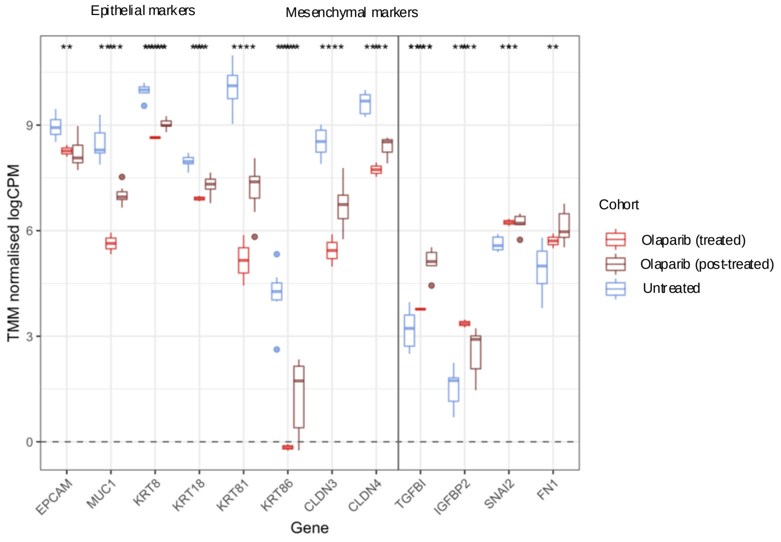

Supplementary Figure 16

A

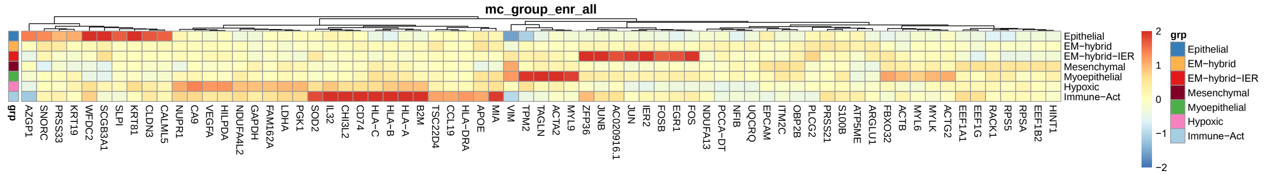

B

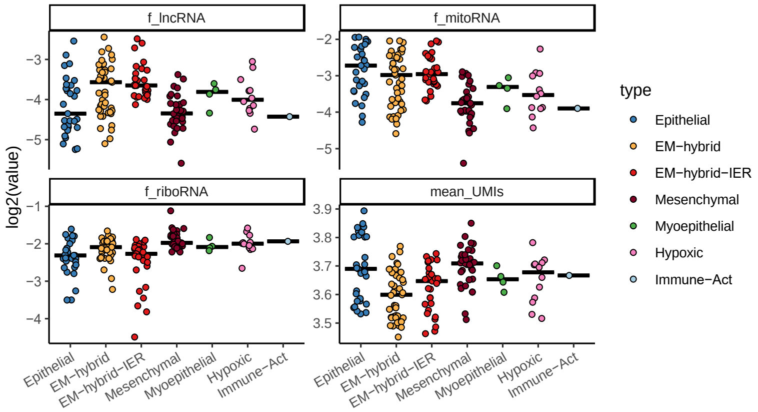

C

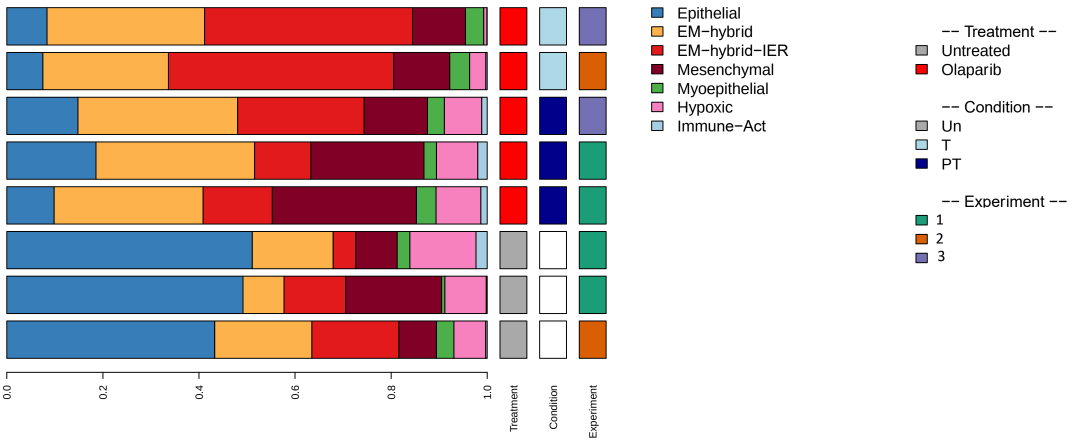

Supplementary Figure 17

A

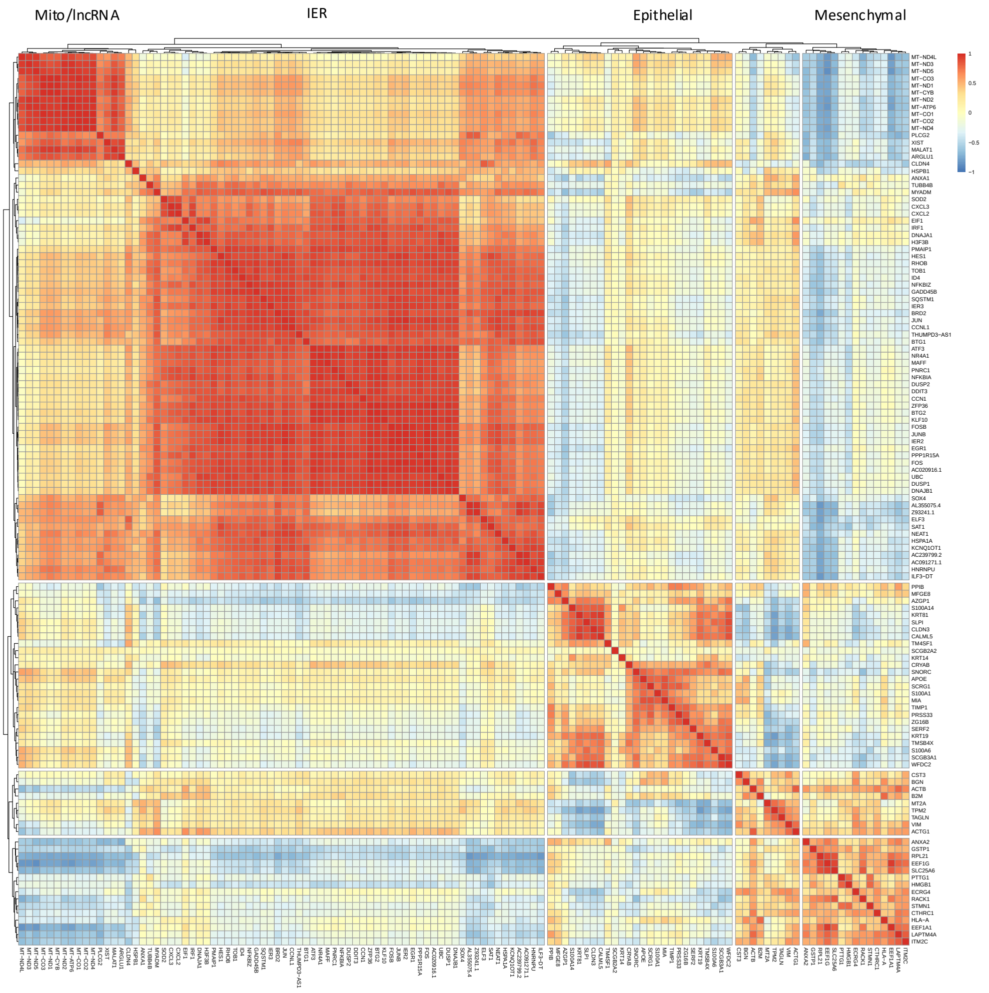

B

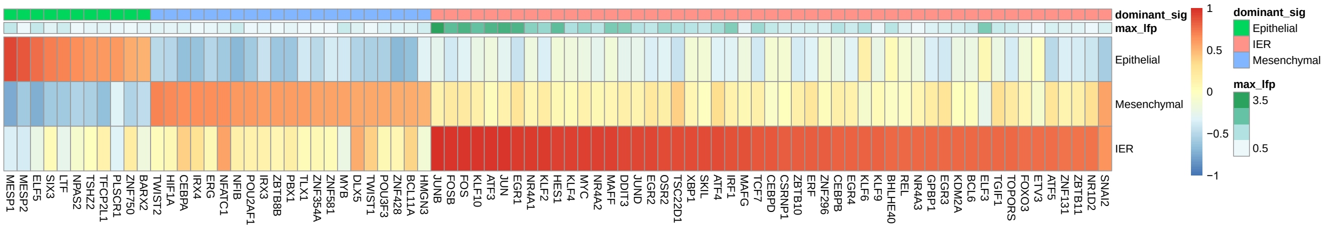

Supplementary Figure 18

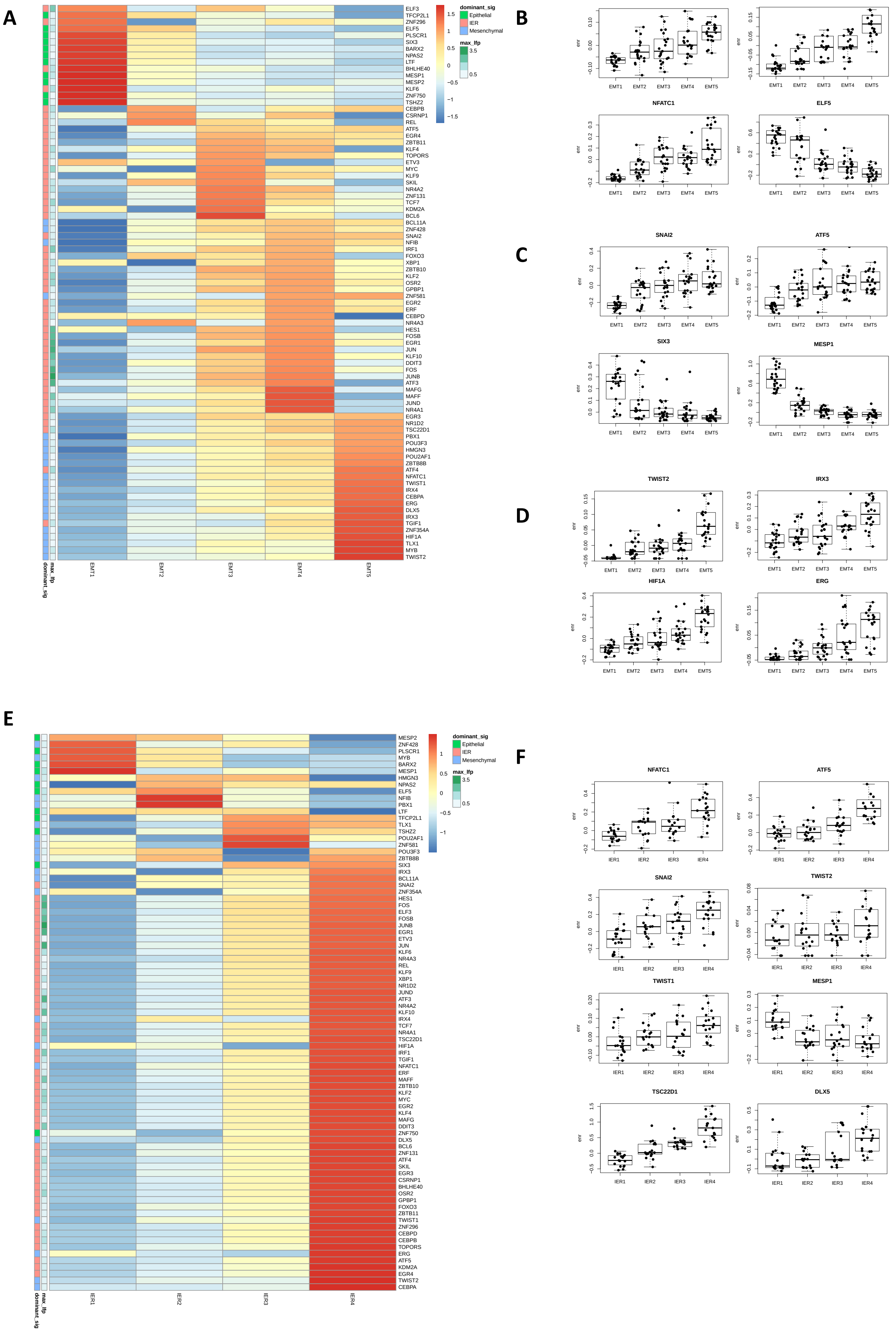

Supplementary Figure 19

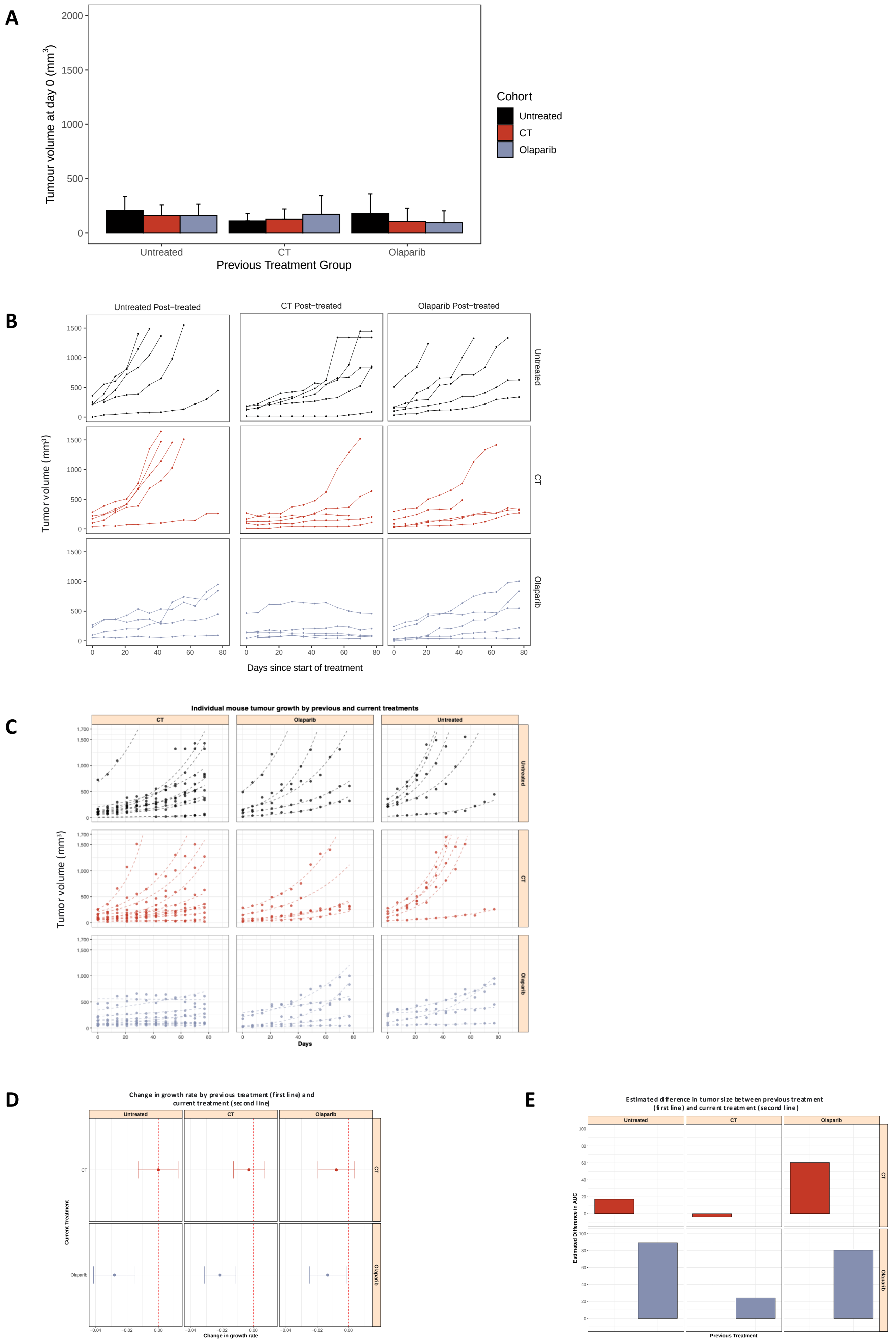

Supplementary Figure 20
