# Supplementary material for: Modeling Drug Responses and Evolutionary Dynamics Using Patient-Derived Xenografts Reveals Precision Medicine Strategies for Triple-Negative Breast Cancer
Source: Cancer Res. 2024 Nov 8;85(3):567–84. doi: 10.1158/0008-5472.CAN-24-1703 (PMC7617242; doi:10.1158/0008-5472.CAN-24-1703)
Supplement: Supplementary Legends — Supplementary Figures 1-20 and Supplementary Tables 1-10 Legends [file can-24-1703_supplementary_legends_suppsd.docx]

**Supplementary Figure 1**

A. Representative immunohistochemistry (IHC) of clinical biomarker expression in PDTX tumors, demonstrating that PDTXs generally retain the expression of the clinical sample from which they were derived. B. Representative IHC demonstrates that PDTX models used in the co-clinical trial were of human, epithelial cell origin.

**Supplementary Figure 2**

A. Principal component analysis of RNA expression data demonstrates that samples separate by PDTX models. B. Heatmap of the Pearson correlation between samples using all genes. Hierarchical clustering performed using ward.d2 method. C-E. Correlation plots comparing variant allele frequencies (VAF) of mutations between multiple regions of the same tumor (C), between sister mice within the same passage (D) and between passage (E). R value calculated using Spearman correlation.

**Supplementary Figure 3**

A-B. Growth curves displaying raw data of untreated and avatar cohorts treated using trial design 1 (A) and trial design 2 (B) over 11 weeks of treatment. C. Percentage change (Δ) in tumor volume curves of avatar cohorts using trial design 2 over 11 weeks of treatment. CT: chemotherapy (paclitaxel and carboplatin). CTO: chemotherapy (paclitaxel and carboplatin) plus Olaparib.

**Supplementary Figure 4**

Analytical metrics derived from the linear mixed models of the co-clinical trial data, for trial design 1 (A-F) and trial design 2 (G-J). A. Box plot displaying the mean difference in daily growth rate between treated and untreated cohorts, estimated with the interaction between growth rate and treatment group in the linear mixed model, for pCR and non-pCR PDTX models. B. Box plot displaying the difference in AUC between the estimated marginal mean growth of the treated and untreated cohorts. C-D. Mean daily growth rates under treatment, as visualized by PDTX model (C) or as a box plot comparing pCR and non-pCR PDTX models (D). E-F. Predicted volume at treatment end, as visualized by PDTX model (E) or as a box plot comparing pCR and non-pCR PDTX models (F). G-H. Mean difference in daily growth rate between treated and untreated cohorts, estimated with the interaction between growth rate and treatment group in the linear mixed model, visualized by PDTX model (G) or as a box plot comparing pCR and non-pCR PDTX models (H). I-J. Difference in AUC between the estimated marginal mean growth of the treated and the untreated groups, visualized by PDTX model (I) or as a box plot comparing pCR and non-pCR PDTX models (J). Statistical significance was tested using Wilcoxon test, comparing pCR and non-pCR PDTX models for each metric.

**Supplementary Figure 5**

Growth curves displaying raw data of models 1040, 1022, 1141, 1006, 1053 and 1008. PDTX models treated with no treatment (untreated), chemotherapy (CT), chemotherapy and Olaparib (CTO), Olaparib, AZD1775 or Olaparib and AZD1775 combination for 11 weeks. PDTX tumors were then allowed to progress off treatment until size limits. End of treatment is indicated by dotted line.

**Supplementary Figure 6**

Metrics derived from the linear mixed modelling, to evaluate response of PDTX models (1006, 1008, 1022, 1040, 1053, 1141) treated with no treatment (untreated), chemotherapy (CT), chemotherapy and Olaparib (CTO), Olaparib, AZD1775 or Olaparib and AZD1775 combination. Metrics describe on-treatment effects of the treatments. A. Mean difference in daily growth rate between treated and untreated cohorts, estimated with the interaction between growth rate and treatment group in the linear mixed model. B. Difference in AUC between the estimated marginal mean growth of the treated and untreated cohorts. C. Mean daily growth rates under treatment. D. Predicted volume at treatment end.

**Supplementary Figure 7**

Linear mixed models to evaluate response of PDTX model 1006 treated with no treatment (untreated), chemotherapy and Olaparib (CTO), Olaparib, AZD1775 or Olaparib and AZD1775 combination. A. Tumor volume growth curves on treatment. B. Tumor volume growth curves post-treatment. C. Mean difference in daily growth rate between treated and untreated cohorts. D. Difference in AUC between the estimated marginal mean growth of the treated and untreated cohorts. E. Mean daily growth rates under treatment. F. Predicted volume at treatment end.

**Supplementary Figure 8**

Linear mixed models to evaluate response of PDTX model 1008 treated with no treatment (untreated), chemotherapy (CT), chemotherapy and Olaparib (CTO), Olaparib, AZD1775 or Olaparib and AZD1775 combination. A. Tumor volume growth curves on treatment. B. Tumor volume growth curves post-treatment. C. Mean difference in daily growth rate between treated and untreated cohorts. D. Difference in AUC between the estimated marginal mean growth of the treated and untreated cohorts. E. Mean daily growth rates under treatment. F. Predicted volume at treatment end.

**Supplementary Figure 9**

Linear mixed models to evaluate response of PDTX model 1022 treated with no treatment (untreated), chemotherapy (CT), chemotherapy and Olaparib (CTO), Olaparib, AZD1775 or Olaparib and AZD1775 combination. A. Tumor volume growth curves on treatment. B. Tumor volume growth curves post-treatment. C. Mean difference in daily growth rate between treated and untreated cohorts. D. Difference in AUC between the estimated marginal mean growth of the treated and untreated cohorts. E. Mean daily growth rates under treatment. F. Predicted volume at treatment end.

**Supplementary Figure 10**

Linear mixed models to evaluate response of PDTX model 1040 treated with no treatment (untreated), chemotherapy (CT), chemotherapy and Olaparib (CTO), Olaparib, AZD1775 or Olaparib and AZD1775 combination. A. Tumor volume growth curves on treatment. B. Tumor volume growth curves post-treatment. C. Mean difference in daily growth rate between treated and untreated cohorts. D. Difference in AUC between the estimated marginal mean growth of the treated and untreated cohorts. E. Mean daily growth rates under treatment. F. Predicted volume at treatment end.

**Supplementary Figure 11**

Linear mixed models to evaluate response of PDTX model 1053 treated with no treatment (untreated), chemotherapy (CT), chemotherapy and Olaparib (CTO), Olaparib, AZD1775 or Olaparib and AZD1775 combination. A. Tumor volume growth curves on treatment. B. Tumor volume growth curves post-treatment. C. Mean difference in daily growth rate between treated and untreated cohorts. D. Difference in AUC between the estimated marginal mean growth of the treated and untreated cohorts. E. Mean daily growth rates under treatment. F. Predicted volume at treatment end.

**Supplementary Figure 12**

Linear mixed models to evaluate response of PDTX model 1141 treated with no treatment (untreated), chemotherapy (CT), chemotherapy and Olaparib (CTO), Olaparib, AZD1775 or Olaparib and AZD1775 combination. A. Tumor volume growth curves on treatment. B. Tumor volume growth curves post-treatment. C. Mean difference in daily growth rate between treated and untreated cohorts. D. Difference in AUC between the estimated marginal mean growth of the treated and untreated cohorts. E. Mean daily growth rates under treatment. F. Predicted volume at treatment end.

**Supplementary Figure 13**

A. Integrative genomics viewer (IGV) of BRCA1 and NRB1 genes in model 1006 and 1040, sequenced using WES. B. Western blot of BRCA1 protein expression in four PDTX models (1006, 1040, 1022, 1141) and cell lines (MDA-MB-231, SUM149, HCC38). C. Normalized gene expression (logCPM) of BRCA1 across PDTX models. D. IGV of BRCA1 in model 1040 displaying the pathogenic mutation (c.4327C>T, p.Arg1443Ter) in untreated, Olaparib treated and Olaparib post-treated samples.

**Supplementary Figure 14**

A. Multi region sequencing approach to interrogate drug response mechanisms to Olaparib. B. Integrative genomics viewer (IGV) to demonstrate Olaparib treated and post-treated tumors from model 1006 retain the homozygous BRCA1 copy number loss observed in the untreated tumor. C. Heatmap displaying VAFs of all exonic, coding, non-synonymous/stop gain SNVs and frameshift indels. Clustering analysis performed using Euclidean distances. T: Treated. PT: Post-treated.

**Supplementary Figure 15**

A. Normalized gene expression (TMM normalized log2CPM) of known PARP inhibitor resistance markers in model 1006 treated *in vivo* with Olaparib. Statistical significance tested between untreated and post-treated tumors using two-tailed unpaired t-test. * p<0.05, ** p<0.01, *** p<0.001, **** p<0.0001. B. Normalized gene expression (TMM normalized log2CPM) of BRCA1 in model 1022 treated *in vivo* with Olaparib. Statistical significance tested using two tailed unpaired t-test. *** p <0.001. C. Differential expression analysis revealed BRCA1 as the most highly differentially expressed gene between untreated and Olaparib post-treated tumors in model 1022 based on log-fold change.

**Supplementary Figure 16**

A. Enrichment plot of HALLMARK EPITHELIAL MESENCHYMAL TRANSITION gene set following GSEA of Olaparib post-treated vs untreated tumors from model 1006. B. Normalized gene expression (TMM normalized log2CPM) of epithelial and mesenchymal markers in model 1006 treated *in vivo* with Olaparib. All genes were found to be differentially expressed between untreated and post-treated samples. Statistical significance tested between untreated and post-treated tumors using two-tailed unpaired t-test. * p<0.05, ** p<0.01, *** p<0.001, **** p<0.0001.

**Supplementary Figure 17**

A. Gene enrichment of representative marker genes for each cell state. Enrichment is capped at +/-2 (log2).  B. Cellular state metrics across the different metacells, showing the fraction of UMIs from lncRNA, mitochondrial and ribosomal genes, and the mean number of UMIs (log2) for cells in each metacell.​ C. Sample cell state composition, including technical replicates.

**Supplementary Figure 18**

A. Gene–gene correlation heatmap of genes over the epithelial, EM-hybrid, EM-hybrid-IER and mesenchymal metacells. Showing genes with minimal enrichment of 1.25 (log2) and a difference of 2.5 between the most and least enriched metacell. Genes are hierarchically clustered, and the tree is cut to 4 clusters. B. Correlation of mildly strong TFs (max enrichment >= 0.2) with the three expression scores. Heatmap displays only TFs with at least 0.5 correlation to one of the scores, ordering TFs by the score they are most correlated with and by their correlation within that group.​

**Supplementary Figure 19**

A. Mean gene enrichment of transcription factors across EMT strata. Epithelial, EM-hybrid, EM-hybrid-IER and mesenchymal metacells were stratified to 5 groups by their mesenchymal minus epithelial scores. Genes are ordered by the strata which they have the highest mean enrichment, and by that enrichment within the strata. B-D. Mean enrichment (log2 gene enrichment score) across EMT strata for genes of interest, which demonstrate step-wise (B), early-acting (C) and late-acting (D) dynamics. E. Mean gene enrichment of transcription factors across IER strata. EM hybrid and EM-hybrid-IER metacells were stratified by IER-score.​ Genes are ordered by the strata which they have the highest mean enrichment, and by that enrichment within the strata. F. Mean enrichment (log2 gene enrichment score) across IER strata for genes of interest.

**Supplementary Figure 20**

A. Bar charts displaying the tumor volume at the start of treatment (day 0) from the preclinical trial testing sequential treatment with CT and Olaparib for model 1040. This indicates that all cohorts started treatment with comparable tumor volumes. Bars show the mean value and error bars display standard deviation. No statistically significant differences were observed between cohorts of the same previous-treatment group, or between previous-treatment groups (tested using unpaired two-tailed t-test, not assuming equal variance). B. Growth curves displaying raw data from the preclinical trial testing sequential treatment with CT and Olaparib for model 1040. C. Linear mixed models of tumor volume growth curves. For both B and C, columns display previous treatment groups (first line treatment) and rows display the cohort of second line treatment. D. Mean difference in daily growth rate between treated and untreated cohorts. Each second line treatment cohort is compared to the untreated cohort within the corresponding previous (first line) treatment group, as displayed per column. E. Difference in AUC between the estimated marginal mean growth of the treated and untreated cohorts. Each second line treatment cohort is compared to the untreated cohort within the corresponding previous (first line) treatment group, as displayed per column.

**Supplementary Table 1**

Genes excluded from single cell RNA-sequencing analysis. This list comprises mitochondrial and a few strong non-coding genes, and gene modules correlated with cell cycle, interferon and stress responses.

**Supplementary Table 2**

Immunohistochemistry (IHC) antibody details.

**Supplementary Table 3**

Immunohistochemistry (IHC) scoring of ER, PR and HER2 from tumor microarray (TMA) cores from multiple mice and passages of each PDTX model used in the study.

**Supplementary Table 4**

Clinical information of PDTX models used for the study.

**Supplementary Table 5**

Top 50 genes correlated with KRT81, VIM and JUNB, used to calculate Epithelial, Mesenchymal and IER expression scores respectively for single-cell RNA-sequencing analysis.
